# Evening-types show highest increase of sleep and mental health problems during the COVID-19 pandemic—multinational study on 19 267 adults

**DOI:** 10.1093/sleep/zsab216

**Published:** 2021-08-25

**Authors:** Ilona Merikanto, Laura Kortesoja, Christian Benedict, Frances Chung, Jonathan Cedernaes, Colin A Espie, Charles M Morin, Yves Dauvilliers, Markku Partinen, Luigi De Gennaro, Yun Kwok Wing, Ngan Yin Chan, Yuichi Inoue, Kentaro Matsui, Brigitte Holzinger, Giuseppe Plazzi, Sérgio Arthuro Mota-Rolim, Damien Leger, Thomas Penzel, Bjørn Bjorvatn

**Affiliations:** 1 SleepWell Research Program Unit, Faculty of Medicine, University of Helsinki, Helsinki, Finland; 2 Department of Public Health Solutions, Finnish Institute for Health and Welfare, Helsinki, Finland; 3 Orton Orthopaedics Hospital, Helsinki, Finland; 4 Centre for Educational Assessment, University of Helsinki, Helsinki, Finland; 5 Department of Neuroscience, Sleep Science (BMC), Uppsala University, Uppsala, Sweden; 6 Department of Anesthesia and Pain Medicine, Toronto Western Hospital, University Health Network, University of Toronto, Toronto, ON, Canada; 7 Institute of Medical Science, Temerty Faculty of Medicine, University of Toronto, Toronto, ON, Canada; 8 Department of Medical Sciences, Uppsala University, Uppsala, Sweden; 9 Department of Medicine, Division of Endocrinology, Metabolism, and Molecular Medicine, Northwestern University, Chicago, IL, USA; 10 Sleep and Circadian Neuroscience Institute, Nuffield Department of Clinical Neurosciences, University of Oxford, Oxford, UK; 11 École de Psychologie, Centre d’étude des troubles du sommeil, Centre de recherche CERVO/Brain Research Center, Université Laval, Québec, QC, Canada; 12 Sleep-Wake Disorders Center, Department of Neurology, Gui-de-Chauliac Hospital, Institute for Neurosciences of Montpellier INM, INSERM, University of Montpellier, Montpellier, France; 13 Helsinki Sleep Clinic, Vitalmed Research Center, and Department of Neurosciences, Clinicum, University of Helsinki, Helsinki, Finland; 14 Department of Psychology, Sapienza University of Rome, Rome, Italy; 15 IRCCS Fondazione Santa Lucia, Rome, Italy; 16 Department of Psychiatry, Faculty of Medicine, The Chinese University of Hong Kong, Shatin, Hong Kong SAR, China; 17 Li Chiu Kong Family Sleep Assessment Unit, Department of Psychiatry, Faculty of Medicine, The Chinese University of Hong Kong, Shatin, Hong Kong SAR, China; 18 Department of Somnology, Tokyo Medical University, Tokyo, Japan; 19 Department of Laboratory Medicine, National Center Hospital, National Center of Neurology and Psychiatry, Tokyo, Japan; 20 Institute for Dream and Consciousness Research, Medical University of Vienna, Vienna, Austria; 21 IRCCS—Institute of the Neurological Sciences of Bologna, Bologna, Italy; 22 Department of Biomedical, Metabolic and Neural Sciences, University of Modena and Reggio Emilia, Modena, Italy; 23 Brain Institute, Physiology and Behaviour Department, and Onofre Lopes University Hospital—Federal University of Rio Grande do Norte, Natal, Brazil; 24 Hopital Hotel-Dieu de Paris, Sleep and Vigilance Center, Universite de Paris, VIFASOM (EA 7331 Vigilance Fatigue Sommeil et Santé Publique), Paris, France; 25 Sleep Medicine Center, Charite Universitätsmedizin Berlin, Berlin, Germany; 26 Department of Global Public Health and Primary Care, University of Bergen, and Norwegian Competence Center for Sleep Disorders, Haukeland University Hospital, Bergen, Norway

**Keywords:** chronotype, circadian rhythms, coronavirus, depression, eveningness, insomnia, sleep quality, stress

## Abstract

**Study Objectives:**

Individual circadian type is a ubiquitous trait defining sleep, with eveningness often associated with poorer sleep and mental health than morningness. However, it is unknown whether COVID-19 pandemic has differentially affected sleep and mental health depending on the circadian type. Here, the differences in sleep and mental health between circadian types are examined globally before and during the COVID-19 pandemic.

**Methods:**

The sample collected between May and August 2020 across 12 countries/regions consisted of 19 267 adults with information on their circadian type. Statistical analyses were performed by using Complex Sample procedures, stratified by country and weighted by the number of inhabitants in the country/area of interest and by the relative number of responders in that country/area.

**Results:**

Evening-types had poorer mental health, well-being, and quality of life or health than other circadian types during the pandemic. Sleep–wake schedules were delayed especially on working days, and evening-types reported an increase in sleep duration. Sleep problems increased in all circadian types, but especially among evening-types, moderated by financial suffering and confinement. Intermediate-types were less vulnerable to sleep changes, although morningness protected from most sleep problems. These findings were confirmed after adjusting for age, sex, duration of the confinement, or socio-economic status during the pandemic.

**Conclusions:**

These findings indicate an alarming increase in sleep and mental health problems, especially among evening-types as compared to other circadian types during the pandemic.

**Statement of Significance**

The current study is unique in examining the effect of the pandemic on sleep problems, sleep schedules, and mental health across 12 countries/regions using a harmonized survey. These multinational findings presented here show an alarming increase in sleep problems amid the pandemic that override any positive effects that the adjustment of sleep–wake behavior towards preferred timing during the pandemic has had. Evening-types showed the highest increase in sleep and mental health problems during the COVID-19 pandemic, such as insomnia, poor sleep quality, nightmares, fatigue, depression, anxiety, and stress. Financial suffering and confinement partly moderated the associations between circadian types and sleep. Based on these findings, there is a real concern to be taken into account in future studies that people with evening circadian type have significantly deteriorated health prospective that has further deteriorated alarmingly during the COVID-19 pandemic. In order to prevent the development of chronic health issues, measures that can alleviate the health risks especially among evening-types are crucial. Examining the benefits of increasing flexibility to societal schedules is of importance to allow better adjustment of the behavioral rhythms according to individual circadian type in the future.

## Introduction

Since the world was faced with the wide spreading of the novel coronavirus from December 2019 onwards, everyday life has been greatly altered due to societal restrictions that have changed social behavior and socioeconomic factors, and shaken our collective sense of security and well-being. Studies from various countries show decreased perceived quality of life ^[1–3]^, increased sleep problems ^[4–7]^, and decreased mental health ^[8]^ during the pandemic. In contrast, “stay at home” and “work from home” restrictions have allowed more flexibility in one’s sleep–wake behavior. For some individuals, confinement has increased sleep duration and enabled the adjustment of bedtimes and wake-up times towards self-desired schedules especially during working days ^[9–11]^. It is important to determine which individuals are most susceptible to impaired sleep and mental health and which individuals might gain benefits from changes in societal schedules during the pandemic in order to evaluate the possible long-term effects on well-being and health in general.

The individual endogenous circadian system is one of the most profound ubiquitous biological mechanisms influencing sleep and mental health ^[12]^. Variation between individuals in their endogenous circadian system includes differences in the timing of behavioral and physiological functions, such as rhythms of sleep–wake, alertness, core body temperature, and hormonal secretion ^[13]^. Based on these differences, individuals can be fitted into a scale of evening- to morning-types ^[14, 15]^. Intra-individually, individual circadian type has been shown to be a fairly persistent trait longitudinally during adulthood ^[16]^. Previous studies have shown that many sleep and mental health problems are commonly observed especially among evening-types ^[17–21]^. As evening-types have a higher vulnerability to impaired health compared to morning- and intermediate-types ^[18]^, it is possible that these health issues are further emphasized among evening-types during the pandemic. However, many of the sleep problems in evening-types arise from attempts to adapt to societal requirements, such as early morning working hours ^[21, 22]^, and so increased flexibility in sleep–wake schedules during the pandemic might actually decrease sleep problems for some people.

No large-scale multinational study has examined the potential differences between circadian types in the emergence of sleep and mental health problems during the COVID-19 pandemic. Previous studies during the COVID-19 pandemic have included rather small samples collected from a few countries and focused on the change in sleep–wake schedule during the pandemic rather than differences between circadian types in sleep and mental health ^[9, 10, 23–25]^, with the exception of one study on Australian athletes ^[26]^. The objective of this multinational study was to examine the potential role of circadian type in explaining sleep and mental health outcomes during the COVID-19 pandemic with a large global sample of 19 267 adults.

## Materials and Methods

### Survey procedure and participants

Altogether 15 countries took part in the International COVID-19 Sleep Study (ICOSS) with a focus on sleep problems before and amid the first wave of the COVID-19 pandemic using a standardized survey. The cross-sectional survey data were collected online in each country in native language between May and August 2020. Ethical approval was granted in each participating country for the study procedure. All procedures performed in studies involving human participants were in accordance with the ethical standards of the institutional and/or national research committee and with the 1964 Helsinki declaration and its later amendments or comparable ethical standards. Informed consent was obtained from all individual participants included in the study. A more detailed description of the survey procedure and items has been published ^[27]^.

Information on circadian type was available from 13 countries/regions: Austria, Brazil, Canada, China/Hong Kong, China/Jinlin, Finland, France, Italy, Japan, Norway, Sweden, UK, and United States. Data from the United States were excluded from this study as the data collection method differed from the other countries resulting in sampling bias towards young men who had suffered financially due to the pandemic and were seeking monetary compensation in participating the study. Participants from other countries did not receive monetary compensation in participating the study. The analytic sample of the present study consisted of 19 267 participants ranging from 18 to 95 years of age (mean age: 41.8 years, SE: 0.1, 65.8% women) with information on circadian type. Supplementary Figure 1 shows the mean age and gender distribution by country.

### Circadian type assessment

Chronotype was assessed with reference to circadian type, which is a commonly used and reliable instrument for describing long-term trends in circadian typology ^[28]^. Circadian type was assessed with a single question “Are you a morning- or evening type-person?” comprising the following response alternatives: (1) I am very alert/active in the morning and sleepy early in the evening (definitively morning-type), (2) I am moderately alert in the morning and sleepy in the evening (moderately morning-type), (3) I am neither morning nor evening person (intermediate type), (4) I am moderately alert in the evening and sleepy in the morning (moderately evening-type), (5) I am very alert/active in the evening and sleepy in the morning (definitively evening-type) ^[29]^.

### Sleep before and amid the first wave of COVID-19 pandemic

General sleep problems, sleep duration per night and per 24 hours, bedtimes, and wake-up times were reported separately for working days and for free days by the participants for both the time before the COVID-19 pandemic (T1) and during the first wave of the pandemic (T2). Sleep behavior was asked separately for the time before the COVID-19 pandemic and for during the pandemic with the following questions: “How many hours per night did/do you sleep on average?” (Reported as hours); “How many hours per 24 hours did/do you sleep on the average (including daytime naps)?” (Reported as hours); “At what time did/do you usually go to bed (to sleep)?” (Asked separately for working days and free days and reported as hours and minutes); “At what time did/do you usually wake up?” (Asked separately for working days and free days and reported as hours and minutes). Participants reporting sleep durations of 0 to 1 hour were excluded from the analyses. Regarding bedtimes, participants reporting later-timed bedtime than their wake-up time were excluded from the analyses. The midpoint of sleep was calculated based on self-reported bedtimes and wake-up times at T1 and T2 to indicate general sleep timing. Midpoint of sleep was determined by the half of the time passed in sleep since going to bed in local time separately for working days and free days ^[30]^.

Sleep problems were asked separately for the time before and during the pandemic with the following questions: (1) Sleep quality: “How well have you been sleeping?” (well, rather well, neither/nor, rather badly, badly); (2) Problems in sleep onset: “Did/do you suffer from difficulty falling asleep?”; (3) Problems in sleep maintenance: “Did/do you suffer from difficulty staying asleep?”; (4) Problems in early morning awakenings: “How often did/do you wake up too early in the morning without being able to fall asleep again?”; (5) Problems in excessive daytime sleepiness: “Did/do you feel excessively sleepy during daytime?”; (6) Problems in daytime fatigue: “Did/do you feel fatigued / exhausted at daytime?” and (7) Nightmares: “How often did/do you have nightmares?” Use of hypnotics was asked separately for before and during the pandemic with the following question: “Did/do you use sleeping pills (hypnotics by prescription)?” Problems in sleep onset, maintenance, early morning awakenings, excessive daytime sleepiness, daytime fatigue, nightmares, and the use of hypnotics were reported as: never or less than once a month, 1–2 times per week, 3–5 times per week, and every or almost every night/day.

The change in sleep behavior and sleep problems between the time before and during the pandemic was calculated as the difference between T1 and T2 (T1–T2), a negative score indicating a decline in sleep problems, sleep duration, or earlier timed bedtimes or wake up times. Participants were divided into groups based on the change in their sleep. Different groups for each sleep item consisted of (1) those whose sleep problem had declined; (2) those whose sleep problem remained unchanged; (3) those whose sleep problem had increased; (4) those whose sleep–wake behavior was earlier-timed; (5) those whose sleep–wake behavior/timing remained unchanged; (6) those whose sleep–wake behavior was later-timed; (7) those whose sleep duration was longer; (8) those whose sleep duration remained unchanged; and (9) those whose sleep duration was shortened amid versus before the pandemic. Habitual daily sleep need was asked with the following question: “How many hours of sleep do you need per day (24 hours)?”

### Insomnia severity index, mental health, and well-being

Severity of insomnia and well-being during the last two weeks before completing the survey were assessed with Insomnia Severity Index (ISI) ^[31]^ and World Health Organization Five Well-Being Index (WHO-5) ^[32]^, respectively. ISI consists of seven questions on insomnia symptoms rated as 0 to 4 on a scale from “none” to “very severe,” respectively ^[31]^. According to the ISI procedure ^[31]^, insomnia severity was assessed here as no insomnia when ISI score ranged from 0 to 7, clinically sub-threshold insomnia when ISI score ranged from 8 to 14, clinically moderate insomnia when ISI score ranged from 15 to 21 and clinically severe insomnia when ISI score ranged from 22 to 28. WHO-5 consists of five statements on feelings/life situations rated from 0 to 5 on a scale from “all the time” to “at no time” ^[32]^. According to the WHO-5 procedure ^[32]^, the raw score multiplied by four giving a scale ranging from 0 to 100, where higher score on the scale indicated higher well-being. Mental health during the last two weeks before completing the survey was assessed separately for anxiety (GAD-2) and for depression (PHQ-2) with The Patient Health Questionnaire for Depression and Anxiety ^[33]^, consisting of four items in total (two assessing anxiety and two assessing depression, rated 0 to 3 on a scale from “not at all” to “nearly every day”). Post-traumatic stress disorder (PTSD) symptoms amid the COVID-19 pandemic were assessed with two items asking about stressful repeated, disturbing memories/thoughts/images and about feeling upset by being reminded of stressful experience (rated 1 to 5 on a scale from “not at all” to “extremely”) ^[34]^. Stress ^[35]^, quality of life, and health at the time of responding to the survey were assessed with single items. Stress was asked about with the following question: “Stress is a condition, in which a person feels tense, troubled, nervous or anxious or sleep is troubled because of things bothering in mind. Do you feel such stress at the moment?” (Rated 1 to 5 on a scale from “not at all” to “very much”). Quality of life and health were asked about separately with a linear visual analog scale from 0 to 100, with 0 indicating the worst possible, and 100 the best possible quality of life/health one could imagine.

### Statistical analyses

All statistical analyses were stratified by country and weighted by the number of inhabitants in the country/area of interest and by the number of responders in that country/area. Complex sample procedures in IBM SPSS Statistics 27 were used with observations as sampling unit for performing the statistical analyses. Complex sample procedures enable sample stratification and weighting in IBM SPSS Statistics, by using an analysis plan file where the sampling unit and commands for specific stratification and weighting measures are defined (https://www.ibm.com/docs/en/spss-statistics/27.0.0?topic=edition-complex-samples). Stratification is important when the data consists of non-overlapping different subgroups of people, such as individuals from a different country (country = strata). Weighting the sample is also necessary when these subgroups differ in size, such as respondent from the specific country/area, and to estimate how representative the subgroup sample is of the strata sample, i.e. taking into consideration the number of inhabitants in the country/area. Differences in sample descriptive information by circadian type were analyzed with chi-square tests within SPSS Complex Samples Crosstabs (CSC) and continuous measurements with t-test within SPSS Complex Samples General Linear Model (CSGLM). Mental health amid the pandemic and sleep before and amid the pandemic by circadian type were analyzed with CSGLM, Complex Samples Logistic Regression (CSLR), or the Complex Samples Ordinal Regression (CSOR). Additional regression analyses adjusted with either duration of the confinement or socio-economic status during the pandemic were also performed to examine the association between circadian type and sleep problems, mental health issues, and well-being amid the pandemic. Moderating roles of confinement and financial suffering due to the pandemic on the association between circadian type and sleep problems amid the pandemic were analyzed with CSOR. Whether circadian type was associated with the change in sleep amid the pandemic was analyzed with the CSOR. All regression analyses were adjusted for gender and age. Definite morning-types were used as a reference group in all the regression analyses. The difference between circadian types in the frequencies of those showing a different direction of sleep behavior change (e.g. increase or decline in sleep problems) or no change was analyzed with chi-square tests within SPSS CSC. The prevalence of being in confinement or having suffered financially among definite evening-types showing decrease/no change or increase in sleep problems was analyzed with chi-square tests within SPSS CSC.

## Results

### Socioeconomic and COVID-19 pandemic related descriptive information by circadian type

Of the analytic sample of 19 267 adults, 11.1% were definite evening-types (*n* = 2135), 23.8% moderate evening-types (*n* = 4589), 26.3% intermediate-types (*n* = 5063), 22.4% moderate morning-types (*n* = 4309), and 16.5% definite morning-types (*n* = 3171). The weighted frequencies of circadian types in the sample were 13.7% (standard error (SE): 0.4%) definite evening-types, 24.8% (SE = 0.5%) moderate evening-types, 22.7% (SE = 0.4%) intermediate-types, 21.9% (SE = 0.4%) moderate morning-types, and 16.9% (SE = 0.4%) definite morning-types. Definite morning-types were 10 years older in their mean age and were better educated than definite evening-types (Table 1, for both age and education *p* < 0.001). Eveningness was more common among women than morningness and vice versa among men (*p* < 0.001).

**Table 1.** Descriptive socioeconomic and pandemic-related information by circadian type

	Definitive evening-types	Moderate evening-types	Intermediate-types	Moderate morning- types	Definitive morning- types	P
Age in years (mean, SE)	33.8 (0.4)	35.2 (0.3)	41.7 (0.3)	39.0 (0.3)	43.8 (0.4)	<0.001
Gender (%)						<0.001
Man	26.2	27.8	36.1	27.5	28.2	
Women	73.8	72.2	63.9	72.5	71.8	
Education (%)						<0.001
Primary/lower 2nd	1.2	1.0	2.3	1.1	2.1	
Secondary/high school	24.3	26.5	24.7	20.0	19.3	
Vocational	8.5	8.5	10.1	9.5	8.6	
Bachelor, RN, B.Sc	40.0	41.4	41.2	41.9	39.9	
Master	18.8	16.9	14.9	19.8	20.5	
Doctoral level MD, PhD	7.2	5.7	6.8	7.6	9.6	
Sleep need in hours per 24 hours	7 h 59 min (0.06)	7 h 46 min (0.03)	7 h 32 min (0.03)	7 h 36 min (0.03)	7 h 26 min (0.05)	<0.001
Present work during pandemic (%)						<0.001
Student	30.8	26.0	16.6	20.0	11.7	
Regular day work	35.6	39.3	45.9	46.8	51.9	
Irregular work	8.9	8.7	7.6	8.4	8.5	
Shift/night work	3.9	4.1	5.0	3.6	3.4	
Unemployed	5.9	6.8	4.6	4.2	4.6	
Retired	3.5	4.5	8.8	7.5	10.4	
At home without salary	6.7	7.0	9.2	7.6	6.6	
Temporarily laid off	1.6	1.6	1.3	1.4	1.5	
Lost job due to pandemic	3.2	2.1	1.0	0.5	1.3	
Suffered financial status due to pandemic (%)						<0.001
Not at all	38.5	36.7	41.8	43.3	44.0	
A little	33.5	35.3	31.2	32.3	32.2	
Somewhat	14.7	15.6	15.1	13.3	13.1	
Much	8.3	8.3	8.2	7.8	6.9	
Very much	5.1	4.0	3.8	3.4	3.8	
Confinement during pandemic (%)						
No	22.0	33.5	52.7	38.2	42.0	<0.001
Yes	78.0	66.5	47.3	61.8	58.0	
Duration of the confinement during pandemic (%)						<0.001
Not been in confinement	22.0	33.5	52.7	38.2	42.0	
≤2 weeks	8.1	9.4	7.5	9.2	8.1	
3 to 4 weeks	6.2	5.5	5.0	5.3	6.4	
5 to 6 weeks	4.3	4.6	3.5	5.0	4.2	
7 to 8 weeks	7.0	7.1	6.0	6.2	6.5	
>8 weeks	52.5	39.9	25.3	36.2	32.8	
Confinement reason						
Confinement due to other reasons than COVID-19 symptoms (Society lockdown, work, travel)	84.7	86.4	88.0	87.1	87.9	
Partly due to lockdown, partly due to COVID-19 symptoms	4.8	5.4	3.2	4.0	2.5	0.5
Due to COVID-19 symptoms in participant or someone in the household	10.5	8.2	8.8	9.0	9.6	
Had COVID-19						
No/don’t know	95.7	96.5	97.1	96.2	94.9	0.06
Yes	4.3	3.5	2.9	3.8	5.1	

Differences between circadian types on categorical descriptive information were analyzed with chi-square tests within SPSS Complex Samples Crosstabs and continuous measurements with *t*-test within SPSS Complex Samples General Linear Model (CSGLM).

In general, habitual daily sleep need was progressively higher among evening-types than among morning-types, with definite evening-types needing on average 33 minutes longer sleep than definite morning-types (*p* < 0.001). The socioeconomic situation during the pandemic was poorer among evening-types than among morning-types with losing job due to the pandemic, unemployment, or studying being more common among evening-types than among morning-types (*p* < 0.001). Evening-types also had a higher risk of suffering financially due to the pandemic compared to morning-types (*p* < 0.001). Frequencies regarding shift/night work, irregular work, or being temporarily laid off did not differ between morning- or evening-types. A greater percentage of evening-types reported having been in confinement and longer periods compared to morning-types (for both *p* < 0.001). There were no significant differences in the self-reported SARS-CoV-2 infection rates or in the reasons for confinement between circadian types (for both infection rate and confinement reasons *p* > 0.05).

### Sleep behavior and sleep problems before and amid the pandemic by circadian type

As shown in Table 2, evening-types slept on average less per night than definite morning-types before the pandemic (for both definite evening-types and for moderate evening-types *p* < 0.001), but no significant differences were observed between evening-types and definite morning-types in the habitual nightly sleep duration during the pandemic. In contrast, during the pandemic, 24-hour sleep duration was significantly longer among evening-types as compared to definite morning-types (for definite evening-types *p* < 0.001 and for moderate evening-types *p* < 0.05). In line with their circadian type, on free days the average bedtimes, wake-up times, and the midpoint of sleep were later-timed for evening-types as compared to definite morning-types both before and during the pandemic (*p* < 0.001 in all the models). Especially for wake-up times, there was a progressive time-difference between the circadian types on both working days and free days, with definite evening-types having the latest and definite morning-types the earliest wake-up times both before and during the pandemic. Amid the pandemic, evening-types had significantly later bedtimes and midpoint of sleep than morning-types also on working days (*p* < 0.001 in all the models). Most results for the associations between circadian type and sleep–wake behavior during the pandemic did not differ significantly between the models adjusted only for age and sex (Table 2) or also for the duration of the confinement or socio-economic status during the pandemic (Supplementary Table 1). Adjusting for duration of the confinement also showed a significantly greater delay in midpoint of sleep on working days and free days not only among evening-types but also among intermediate-types (for both working days and free days *p* < 0.05) as compared to definite morning-types. Adjusting for socio-economic status during the pandemic indicated that also moderate morning-types had later bedtimes on free days (*p* < 0.05) as compared to definite morning-types amid the pandemic.

**Table 2.** Sleep behavior before vs. amid the pandemic by circadian type

	Definitive evening-types	Moderate evening-types	Intermediate-types	Moderate morning-types	Definite morning-types
Sleep per night in hours					
Before pandemic (Mean hh.mm)	6 h 50 min	6 h 53 min	6 h 52 min	7 h 5 min	7 h 4 min
Before pandemic (B, 95% CI)	–0.2 (–0.3 ± –0.1)***	–0.2 (–0.3 ± –0.1)***	–0.2 (–0.3 ± –0.1)***	0.02 (–0.06 ± 0.1)	
During pandemic (Mean hh.mm)	7 h 3 min	7 h 7 min	6 h 59 min	7 h 13 min	7 h 5 min
During pandemic (B, 95% CI)	–0.03 (–0.2 ± 0.1)	0.03 (–0.08 ± 0.1)	–0.09 (–0.02 ± 0.01)	0.1 (0.04 ± 0.3)**	
Change (Mean hh.mm)	+13 min	+14 min	+7 min	+8 min	+1 min
Sleep per 24 h in hours					
Before pandemic (Mean hh.mm)	7 h 20 min	7 h 14 min	7 h 10 min	7 h 22 min	7 h 21 min
Before pandemic (B, 95% CI)	–0.02 (–0.2 ± 0.1)	–0.1 (–0.2 ± –0.01)*	–0.2 (–0.3 ± –0.1)***	0.02 (–0.08 ± 0.1)	
During pandemic (Mean hh.mm)	7 h 57 min	7 h 37 min	7 h 24 min	7 h 34 min	7 h 28 min
During pandemic (B, 95% CI)	0.5 (0.3 ± 0.7)***	0.2 (0.02 ± 0.3)*	–0.06 (–0.2 ± 0.2)	0.1 (–0.02 ± 0.2)	
Change (Mean hh.mm)	+37 min	+23 min	+14 min	+12 min	+7 min
Bedtime on working days					
Before pandemic (Mean hh:mm)	1:17	0:51	0:42	0:23	1:02
Before pandemic (B, 95% CI)	909.5 (–686.6 ± 2505.6)	–652.8 (–2210.0 ± 904.4)	–1218.3 (–2847.2 ± 410.7)	–2323.0 (–3887.7 ± –758.3)**	
During pandemic (Mean hh:mm)	2:24	1:29	1:09	0:56	1:02
During pandemic (B, 95% CI)	4902.2 (3996.8 ± 5807.6)***	1608.3 (762.8 ± 2453.7)***	387.2 (–525.0 ± 1299.3)	–367.3 (–1258.5 ± 523.9)	
Change (Mean hh:mm)	+1 h 7 min	+38 min	+27 min	+33 min	0 min
Wake-up time at working days					
Before pandemic (Mean hh:mm)	7:35	7:05	6:49	6:33	6:21
Before pandemic (B, 95% CI)	4473.4 (3963.8 ± 4983.1)***	2655.6 (2262.5 ± 3048.7)***	1707.2 (1324.6 ± 2089.8)***	730.7 (363.3 ± 1098.0)***	
During pandemic (Mean hh:mm)	9:02	8:04	7:25	7:11	6:50
During pandemic (B, 95% CI)	7932.4 (7217.1 ± 8647.8)***	4430.0 (3954.1 ± 4906.0)***	2121.8 (1652.2 ± 2591.4)***	1260.3 (809.9 ± 1710.7)***	
Change (Mean hh:mm)	+1 h 27 min	+59 min	+36 min	+38 min	+29 min
Bedtime on free days					
Before pandemic (Mean hh:mm)	1:45	1:13	0:52	0:38	0:46
Before pandemic (B, 95% CI)	3559.5 (2765.9 ± 4353.9)***	1593.5 (958.4 ± 2228.5)***	355.9 (–257.1 ± 986.8)	–451.4 (–1115.5 ± 212.8)	
During pandemic (Mean hh:mm)	2:34	1:42	1:13	1:02	1:15
During pandemic (B, 95% CI)	4749.4 (3926.3 ± 5572.4)***	1652.8 (916.2 ± 2389.4)***	–98.0 (–848.1 ± 652.1)	–767.0 (–1561.5 ± 27.5)	
Change (Mean hh:mm)	+49 min	+29 min	+21 min	+24 min	+29 min
Wake-up time on free days					
Before pandemic (Mean hh:mm)	9:34	8:52	8:03	7:51	7:30
Before pandemic (B, 95% CI)	7443.0 (6760.3 ± 8125.6)***	4921.2 (4417.6 ± 5424.7)***	2009.4 (1527.6 ± 2491.2)***	1268.9 (786.4 ± 1751.4)***	
During pandemic (Mean hh:mm)	10:06	9:07	8:15	8:02	7:34
During pandemic (B, 95% CI)	9137.9 (8417.4 ± 9858.5)***	5579.9 (5069.2 ± 6990.6)***	2450.2 (1948.9 ± 2951.4)***	1696.3 (1174.8 ± 2217.8)***	
Change (Mean hh:mm)	+32 min	+15 min	+12 min	11 min	+4 min
Midpoint of sleep at working days					
Before pandemic (Mean hh:mm)	4:44	4:12	3:59	3:45	4:14
Before pandemic (B, 95% CI)	1771.9 (259.7 ± 3284.1)*	–114.8 (–1585.7 ± 1356.1)	–878.1 (–2410.9 ± 654.6)	–1740.3 (–3224.0 ± –256.7)*	
During pandemic (Mean hh:mm)	6:05	5:08	4:41	4:32	4:29
During pandemic (B, 95% CI)	5799.1 (4896.8 ± 6701.4)***	2336.0 (1506.2 ± 3165.9)***	705.5 (–190.1 ± 1601.1)	195.3 (–674.8 ± 1065.3)	
Change (Mean hh:mm)	+1 h 21 min	+56 min	+40 min	+47 min	+15 min
Midpoint of sleep at free days					
Before pandemic (Mean hh:mm)	5:58	5:18	4:45	4:36	4:40
Before pandemic (B, 95% CI)	4715.8 (3969.1 ± 5462.5)***	2274.1 (1657.8 ± 2890.4)***	296.1 (–336.1 ± 928.3)	–237.3 (–877.1 ± 402–6)	
During pandemic (Mean hh:mm)	6:40	5:43	5:05	4:56	4:58
During pandemic (B, 95% CI)	6152.7 (5420.1 ± 6885.3)***	2751.1 (2121.6 ± 3380.6)***	456.7 (–198.3 ± 1111.6)	–75.9 (–761.8 ± 610.1)	
Change (Mean hh:mm)	+42 min	+25 min	+20 min	+20 min	+18 min

Definite morning-types are defined as reference in the regression models, adjusted for age and sex. Mean (hh:mm), regression estimate (B) and confidence intervals (95% CI) given in the table. ***p < 0.001, **p < 0.01, *p < 0.05.

As shown in Table 3, a progressive difference in most of the sleep problems was seen between circadian types, with definite evening-types having the most and definite morning-types the least problems regarding fatigue, sleep quality, sleep onset, excessive sleepiness, and nightmares both before and during the pandemic (*p* < 0.001 for all these sleep problems). Evening-types, and especially definite evening-types, also had more sleep maintenance problems (for definite evening-types *p* < 0.05 before the pandemic and *p* < 0.01 during the pandemic; for moderate evening-types *p* < 0.05 before the pandemic) and used more hypnotics (for definite evening-types *p* < 0.001 in all the models) both before and during the pandemic as compared to definite morning-types. In line with their circadian type, definite morning-types reported significantly more early morning awakenings both before and amid the pandemic than evening- or intermediate-types (*p* < 0.01 to *p* < 0.001). Lastly, eveningness was associated with higher insomnia severity during the pandemic (*p* < 0.001 in all the models), while intermediate-types had lower insomnia severity than definite morning-types (*p* < 0.01). The prevalence of clinically severe insomnia was 3 to 5 times higher among definite evening-types than among morning- or intermediate-types. The results on the associations between circadian type and sleep problems during the pandemic did not for the most part differ significantly between the models adjusted only for age and sex (Table 3) or also for the duration of the confinement or socio-economic status during the pandemic (Supplementary Table 1). Adjusting for the duration of the confinement also indicated significantly more excessive sleepiness not only among evening-types but also among intermediate-types (*p* < 0.01) as compared to definite morning-types and there was no longer a significant difference in the insomnia severity between intermediate-types and definite morning-types.

**Table 3.** Sleep problems before and amid the pandemic by circadian type

	Definitive evening-types	Moderate evening-types	Intermediate-types	Moderate morning-types	Definite morning-types
Poor sleep quality					
Before pandemic (%)^a^	21.0	15.6	13.9	11.3	11.8
Before pandemic (B, 95% CI)	0.8 (0.6 ± 0.9)***	0.6 (0.5 ± 0.7)***	0.6 (0.5 ± 0.7)***	0.2 (0.1 ± 0.4)***	
During pandemic (%)^a^	43.2	31.6	27.5	26.7	27.4
During pandemic (B, 95% CI)	0.8 (0.6 ± 0.9)***	0.4 (0.3 ± 0.5)***	0.2 (0.1 ± 0.3)***	0.1 (0.008 ± 0.3)*	
Sleep onset problems					
Before pandemic (%)^b^	29.7	19.4	12.7	8.9	9.1
Before pandemic (B, 95% CI)	1.3 (1.2 ± 1.5)***	0.9 (0.7 ± 0.9)***	0.4 (0.3 ± 0.6)***	0.2 (0.1 ± 0.4)***	
During pandemic (%)^b^	53.1	37.1	24.7	20.9	20.5
During pandemic (B, 95% CI)	1.4 (1.2 ± 1.5)***	0.8 (0.7 ± 0.9)***	0.3 (0.1 ± 0.4)***	0.2 (0.06 ± 0.3)**	
Sleep maintenance problems					
Before pandemic (%)^b^	18.1	15.7	18.1	15.3	18.3
Before pandemic (B, 95% CI)	0.2 (0.05 ± 0.4)*	0.1 (0.006 ± 0.3)*	0.07 (–0.05 ± 0.2)	0.04 (–0.08 ± 0.2)	
During pandemic (%)^b^	34.3	27.9	27.0	25.3	29.7
During pandemic (B, 95% CI)	0.2 (0.09 ± 0.4)**	0.06 (–0.06 ± 0.2)	–0.1 (–0.2 ± –0.0003)*	–0.004 (–0.1 ± 0.1)	
Early morning awakening					
Before pandemic (%)^b^	11.8	9.9	10.2	10.4	16.5
Before pandemic (B, 95% CI)	–0.2 (–0.4 ± –0.05)**	–0.3 (–0.4 ± –0.2)***	–0.3 (–0.4 ± –0.2)***	–0.1 (–0.2 ± 0.02)	
During pandemic (%)^b^	20.2	17.2	16.9	19.5	26.4
During pandemic (B, 95% CI)	–0.3 (–0.4 ± –0.1)***	–0.3 (–0.4 ± –0.1)***	–0.4 (–0.5 ± –0.3)***	–0.1 (–0.2 ± 0.02)	
Hypnotics use					
Before pandemic (%)^b^	7.3	5.8	5.1	3.1	4.1
Before pandemic (B, 95% CI)	0.7 (0.5 ± 0.9)***	0.2 (–0.007 ± 0.4)	0.1 (–0.09 ± 0.4)	–0.07 (–0.3 ± 0.2)	
During pandemic (%)^b^	10.1	7.2	6.2	4.7	6.4
During pandemic (B, 95% CI)	0.5 (0.3 ± 0.7)***	0.08 (–0.1 ± 0.3)	–0.02 (–0.2 ± 0.2)	–0.1 (–0.4 ± 0.07)	
Excessive sleepiness					
Before pandemic (%)^b^	30.6	21.6	16.9	13.2	12.0
Before pandemic (B, 95% CI)	1.1 (0.9 ± 1.2)***	0.7 (0.6 ± 0.9)***	0.3 (0.2 ± 0.4)***	0.3 (0.1 ± 0.4)***	
During pandemic (%)^b^	44.8	30.4	22.4	18.6	20.4
During pandemic (B, 95% CI)	1.1 (0.9 ± 1.2)***	0.6 (0.5 ± 0.7)***	0.1 (–0.01 ± 0.2)	0.1 (0.02 ± 0.3)*	
Fatigue					
Before pandemic (%)^b^	32.5	24.9	19.2	17.4	16.8
Before pandemic (B, 95% CI)	0.8 (0.7 ± 0.9)***	0.6 (0.4 ± 0.7)***	0.2 (0.08 ± 0.3)***	0.2 (0.9 ± 0.3)***	
During pandemic (%)^b^	47.2	33.3	25.1	24.7	25.1
During pandemic (B, 95% CI)	0.9 (0.7 ± 1.0)***	0.4 (0.3 ± 0.6)***	–0.03 (–0.1 ± 0.09)	0.07 (–0.05 ± 0.2)	
Nightmares					
Before pandemic (%)^b^	6.6	4.8	4.0	3.0	3.7
Before pandemic (B, 95% CI)	0.5 (0.3 ± 0.6)***	0.3 (0.2 ± 0.5)***	0.2 (0.07 ± 0.3)**	0.2 (0.07 ± 0.3)**	
During pandemic (%)^b^	18.7	11.9	8.9	8.1	8.2
During pandemic (B, 95% CI)	0.6 (0.4 ± 0.8)***	0.3 (0.2 ± 0.4)***	0.06 (–0.07 ± 0.2)	0.1 (–0.02 ± 0.2)	
Insomnia severity index (ISI) amid pandemic					
Moderately severe insomnia (ISI: 15 to 21) (%)	26.5	19.8	12.9	14.7	16.8
Clinically severe insomnia (ISI≥22) (%)	11.9	4.9	4.4	2.2	4.3
ISI (B, 95% CI)	0.9 (0.8 ± 1.1)***	0.3 (0.2 ± 0.4)***	–0.2 (–0.3 ± –0.04)**	–0.07 (–0.2 ± 0.07)	

Definite morning-types are defined as reference in the regression models, adjusted for age and sex. ***p < 0.001, **p < 0.01,*p < 0.05. The combined percentage of ^a^ those sleeping “rather badly” or “badly” and ^b^ those reporting sleep problems ≥3 days/nights per week.

Financial suffering due to the pandemic and confinement moderated the associations between circadian type and sleep problems amid the pandemic (Supplementary Table 2). Sleep problems were pronounced among those who had suffered financially or been in confinement during the pandemic. Sleep problems were emphasized among definite evening-types as compared to other circadian types, regardless of whether or not they had suffered financially or been in confinement, but especially when they had.

### Worsening and improvement of sleep during the pandemic by circadian type

In general, both nightly and 24-hour sleep duration increased among all circadian types amid the pandemic compared to before, but the increase was emphasized among evening-types and especially in definite evening-types (Table 2). Similarly, the delay during the pandemic in bedtimes and wake-up times both on working and free days was greater among evening-types and especially among definite evening-types and more prominent on working days than on free days.

**Figure 1.**
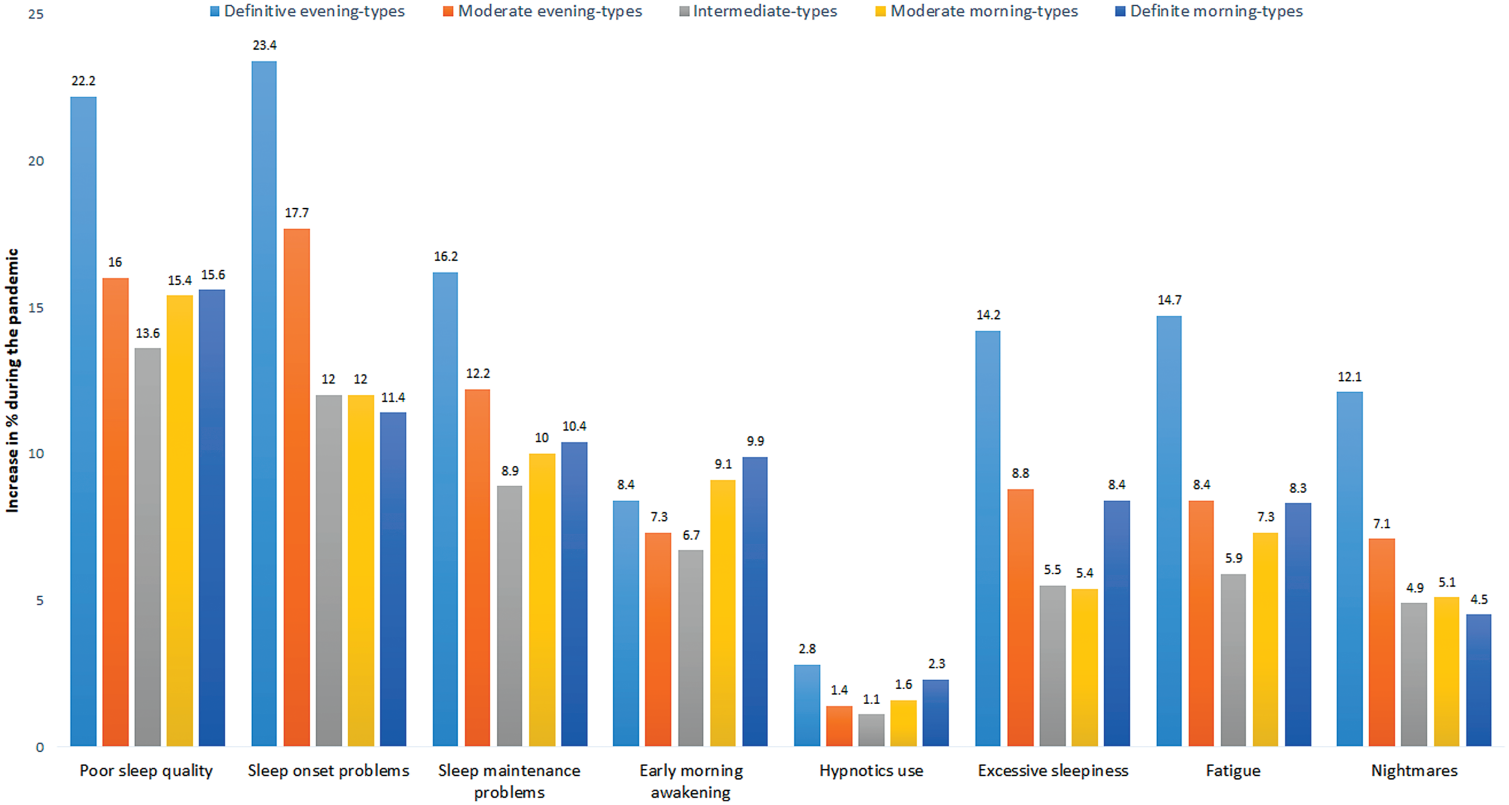
Worsening of sleep and daytime function from before to amid the pandemic by circadian type. Figure shows for poor sleep quality the increase amid pandemic of those sleeping “rather badly” or “badly” and for other sleep problems the increase amid pandemic of those reporting sleep problems ≥3 days/nights per week.

All sleep problems increased amid the pandemic among all circadian types, but especially among the definite evening-types. Only the prevalence of early morning awakenings increased the most among the definite morning-types (Figure 1). Of all the sleep problems, sleep onset problems and poor sleep quality showed the most marked increase among the circadian types during the pandemic. Of the circadian types, the definite morning-types showed the least increase in nightmares. For other sleep problems, the intermediate-types showed the least increase in sleep problems of all the circadian types.

Although the general trend during the pandemic was an increase in sleep problems, longer sleep duration, and a delay in sleep–wake timing, some individuals reported less sleep problems and shorter sleep duration, and an earlier-timed sleep–wake rhythm (Table 4). For most evening-types, the bedtimes, wake-up times, and midpoint of sleep were delayed both on working days and on free days. The delay in sleep–wake behavior, on the other hand, was less apparent among morning-types, especially among the definite morning-types as compared to other circadian types. Among definite morning-types, the shift towards earlier-timed sleep–wake behavior during the pandemic was generally more common than among other circadian types, especially on free days. Definite evening-types had the highest prevalence of both those reporting an increase and a decrease in nightly sleep duration during the pandemic. Similarly, of all the circadian types, definite evening-types had the highest prevalence of those with either an increase or a decrease in sleep maintenance problems, early morning awakening, hypnotic use, excessive sleepiness, fatigue, and nightmares. This suggests that among evening-types there were fewer who reported unchanged sleep patterns as compared to the other circadian types (p<0.001). Supplementary Table 3 shows more detailed differences regarding confinement and financial status among definite evening-types by changes in sleep problems. Confinement was more common among definite evening-types showing either a decrease or especially an increase, rather than no change at all in sleep problems amid the pandemic (for sleep problems *p* < 0.001, *p* < 0.05 for hypnotics use). Change in financial status amid the pandemic, on the other hand, did not differ as much as being in confinement regarding change in sleep problems among definite evening-types (for nightly sleep and for nightmare *p* < 0.01). The definite evening-types had the highest variability in the occurrence of sleep problems during the pandemic in relation to before the pandemic (baseline) as compared to other circadian types. The prevalence of baseline sleep problems was highest among definite evening-types showing a decrease in sleep problems during the pandemic, while the opposite was true for definite evening-types showing an increase in sleep problems during the pandemic. All in all, sleep problems increased amid the pandemic to even higher frequencies than seen among definite evening-types before the pandemic (Supplementary Table 3).

**Table 4.** Change in sleep shown as percentage (%) of those reporting decrease, no change, or increase

	Definitive evening-types	Moderate evening-types	Intermediate-types	Moderate morning-types	Definitive morning-types	P^a^
Sleep per night in hours						<0.001
Decreased	31.2	28.3	22.8	26.6	27.9	
Unchanged	23.4	31.4	47.8	40.8	46.7	
Increased	45.4	40.3	29.4	32.6	25.5	
Sleep per 24 h in hours						<0.001
Decreased	25.3	25.4	24.8	27.5	28.7	
Unchanged	25.3	31.2	44.7	38.7	43.7	
Increased	49.3	43.4	30.6	33.8	27.5	
Bedtime on working days						<0.001
Earlier	9.5	9.8	8.5	7.4	10.4	
Unchanged	24.2	38.6	58.0	48.7	53.1	
Later	66.3	51.6	33.4	43.9	36.5	
Wake-up time at working days						<0.001
Earlier	14.6	13.6	15.4	17.1	19.9	
Unchanged	19.2	25.2	39.3	31.7	40.2	
Later	66.2	61.1	45.3	51.2	39.9	
Bedtime on free days						<0.001
Earlier	9.7	10.9	11.1	11.1	12.3	
Unchanged	33.4	40.0	56.4	48.3	50.9	
Later	56.8	49.1	32.5	40.6	36.8	
Wake-up time on free days						<0.001
Earlier	20.5	21.5	19.4	22.5	22.9	
Unchanged	30.7	38.3	52.7	45.2	49.1	
Later	48.8	40.2	28.0	32.3	28.0	
Midpoint of sleep at working days						<0.001
Earlier	11.5	12.3	12.1	11.5	12.8	
Unchanged	15.2	26.2	40.3	32.5	42.3	
Later	73.3	61.5	47.6	56.0	44.9	
Midpoint of sleep at free days						<0.001
Earlier	18.8	21.8	16.3	20.2	20.5	
Unchanged	21.2	30.2	46.4	36.6	36.1	
Later	60.0	48.0	37.3	43.2	43.4	
Poor sleep quality						<0.001
Decreased	15.8	16.2	11.7	13.4	10.9	
Unchanged	36.5	41.9	58.6	46.7	50.5	
Increased	47.7	42.0	29.7	39.9	38.6	
Sleep onset problems						<0.001
Decreased	11.9	12.8	9.4	10.4	9.0	
Unchanged	43.0	45.1	62.2	53.6	58.3	
Increased	45.2	42.1	28.4	36.0	32.7	
Sleep maintenance problems						<0.001
Decreased	10.2	9.6	8.8	9.8	9.1	
Unchanged	53.1	57.3	66.3	56.9	58.7	
Increased	36.7	33.1	24.9	33.3	32.2	
Early morning awakening						<0.001
Decreased	13.9	11.9	8.7	10.3	10.7	
Unchanged	55.0	57.1	69.0	59.5	59.9	
Increased	31.1	30.9	22.3	30.2	29.4	
Hypnotics use						<0.001
Decreased	3.9	1.7	2.2	1.8	2.0	
Unchanged	86.5	92.1	92.3	92.2	90.6	
Increased	9.6	6.2	5.5	6.0	7.4	
Excessive sleepiness						<0.001
Decreased	19.9	19.0	13.7	16.8	12.3	
Unchanged	43.9	48.7	63.7	53.1	58.6	
Increased	36.3	32.3	22.6	30.1	29.1	
Fatigue						<0.001
Decreased	21.3	19.8	15.8	19.2	13.6	
Unchanged	39.6	46.3	59.8	50.3	55.2	
Increased	39.1	33.9	24.3	30.5	31.2	
Nightmares						<0.001
Decreased	7.0	5.8	5.3	6.3	5.2	
Unchanged	54.3	62.5	73.1	66.7	68.9	
Increased	38.7	31.8	21.6	27.0	25.9	

^a^ Difference between circadian types were analyzed with chi-square tests.

### Mental health during the pandemic by circadian type

Mental health status amid the pandemic was significantly worse among evening-types and especially among definite evening-types as compared to morning-types (Table 5). More evening-types reported symptoms of depression than definite morning-types (*p* < 0.001 for both moderate and definite evening-types). Definite evening-types also reported more anxiety symptoms than definite morning-types (*p* < 0.001), while fewer intermediate-types (*p <* 0.001) and moderate morning-types (*p* < 0.05) reported anxiety than definite morning-types. The prevalence of having depressive or anxiety symptoms among definite evening-types was also higher than the prevalence of not being depressed or not having anxiety. Furthermore, evening-types had more stress (*p* < 0.001 for both moderate and definite evening-types), repeated disturbing thoughts and memories (*p* < 0.001 for both moderate and definite evening-types), and feeling upset about the past (*p* < 0.001 for both moderate and definite evening-types) than definite morning-types. Well-being index was lower in all the other circadian types (*p* < 0.001 for all circadian types) as compared to definite morning-types, with definite evening-types having the lowest well-being index. Lastly, a progressive difference in the quality of life and the quality of health was seen between circadian types, with definite evening-types reporting the worse quality of life (*p* < 0.001) and health (*p* < 0.001) as compared to definite morning-types. The results on the associations between circadian type and mental health during the pandemic did not differ significantly between the models adjusted only for age and sex (Table 5) or for the duration of the confinement or socio-economic status during the pandemic (Supplementary Table 1). Financial suffering and confinement due to the pandemic moderated the associations between circadian types and mental health amid the pandemic (Supplementary Table 4). Mental health problems were emphasized among definite evening-types as compared to other circadian types (*p* < 0.001 in all the models as compared to definite morning-types), regardless of whether or not they had suffered financially or been in confinement, but especially when they had. Definite morning-types who had not suffered financially or been in confinement had the least mental health problems of all the circadian types, with the only exception of anxiety being higher among definite morning-types than among intermediate-types who had not suffered financially during the pandemic.

**Table 5.** Mental health amid the pandemic by circadian type

	Definitive evening-types			Moderate evening-types			Intermediate-types			Moderate morning-types		
	Mean/%	B	95% CI Lower ± Upper	Mean/%	B	95% CI Lower ± Upper	Mean/%	B	95% CI Lower ± Upper	Mean/%	B	95% CI Lower ± Upper
Anxiety past two weeks^a^	54.4	0.6	0.4 ± 0.8***	43.0	0.1	–0.02 ± 0.3	28.6	–0.3	–0.4 ± –0.1***	33.2	–0.2	–0.4 ± –0.04*
Depression past two weeks^a^	50.5	0.9	0.7 ± 1.1***	36.6	0.3	0.2 ± 0.5***	24.3	–0.07	–0.2 ± 0.1	27.8	0.04	–0.1 ± 0.2
Well-being index past two weeks	44.2	–15.0	–16.8 ± –13.2***	50.3	–8.9	–10.5 ± –7.4***	56.1	–3.2	–4.7 ± –1.7***	55.8	–3.5	–5.0 ± –1.9***
PTSD symptoms amid pandemic: Repeated disturbing thoughts and memories^b^	30.0	0.8	0.6 ± 1.0***	19.8	0.3	0.2 ± 0.4***	13.4	0.03	–0.09 ± 0.2	12.8	0.03	–0.09 ± 0.2
PTSD symptoms amid pandemic: Feeling very upset of past^b^	30.7	0.8	0.6 ± 0.9***	20.2	0.3	0.1 ± 0.4***	13.5	0.02	–0.1 ± 0.1	13.8	0.02	–0.1 ± 0.2
Stress at the time of response^c^	45.0	0.9	0.7 ± 1.0***	31.8	0.4	0.2 ± 0.5***	22.6	–0.04	–0.2 ± 0.08	23.5	0.08	–0.04 ± 0.2
Quality of life at the time of response	58.4	–8.4	–10.3 ± –6.5***	64.0	–2.9	–4.3 ± –1.4***	64.3	–2.5	–3.9 ± –1.1***	67.2	0.3	–2.2 ± 1.8
Quality of health at the time of response	64.8	–7.2	–9.1 ± –5.2***	68.5	–3.5	–5.0 ± –2.0***	69.8	–2.2	–3.6 ± –0.7**	71.7	–0.3	–1.7 ± 1.2

Definite morning-types are defined as reference in the regression models, adjusted for age and sex. Table shows in the % column the prevalence of ^a^ those with anxiety/depression with no anxiety and no depression as reference category in the logistic regression, ^b^ those reporting PTSD symptoms “quite a bit” or “extremely” and ^c^ reporting stress “quite a lot” or “very much.” ***p < 0.001, **p < 0.01, *p < 0.05. Regression estimate (B) and confidence intervals (95% CI) given in the Table. PTSD refers to post-traumatic stress disorder.

## Discussion

This study employs a large multinational sample to examine whether the COVID-19 pandemic has had different effects on sleep and mental health depending on individual circadian type. In line with previous studies on the associations between sleep problems and eveningness ^[18, 19]^, evening-types suffered more sleep problems, such as poor sleep quality, problems with sleep onset and maintenance, excessive sleepiness, daytime fatigue, nightmares, and more hypnotics use than other circadian types both before and amid the pandemic. Morning-types, in contrast, showed the least sleep problems both before and during the pandemic versus intermediate- or evening-types. An exception to this general pattern was the higher prevalence of early morning awakenings among the definite morning-types, which on the other hand might have reflected more the innate morningness in sleep–wake rhythms rather than a sleep problem. Despite having longer sleep durations, which was mainly seen among evening-types, the prevalence of sleep problems increased among all the circadian types from before to during the pandemic, but these changes were especially pronounced among evening-types. Of the circadian types, intermediate-types showed the least amount of change in sleep problems amid the pandemic, indicating that the more extreme circadian types were more vulnerable to sleep changes, although morningness seems to protect from most of the sleep problems in general. The most marked increase in sleep problems was reported for sleep onset problems and for poor sleep quality amid the pandemic. Increase in sleep problems amid the pandemic have also been reported in previous smaller general public samples from some countries ^[4, 5, 7, 9]^. Financial suffering due to the pandemic and confinement partly moderated the prevalence of sleep problems among all circadian types amid the pandemic, elevating these problems among those who had suffered financially or been in confinement. These findings are in line with a previous study showing an association between confinement and increased sleep problems ^[36]^. It is likely that not only lockdown restrictions, but also other factors related to the pandemic, such as increased stress, have had a negative effect on sleep and wellbeing as, based on our findings, financial suffering and confinement do not completely explain the increase in sleep problems amid the pandemic.

Along with higher prevalence of sleep problems and higher insomnia severity, evening-types reported poorer mental health during the pandemic than other circadian types, such as more symptoms of depression and anxiety, post-traumatic stress symptoms, stress, and poorer general well-being, quality of life and health amid the pandemic. Definite morning-types, on the other hand, evaluated their well-being and quality of life and health to be better than other circadian types. As sleep, stress, and mental health are often intertwined ^[37, 38]^ and evening-types typically have more impairment in sleep and mental health compared to other circadian types ^[18, 20, 21]^, higher levels of these health problems would be expected especially among evening-types than among other circadian types at a stressful time such as the pandemic. The socioeconomic situation among the evening-types was also much poorer amid the pandemic versus other circadian types, which may interact with the risk for mood and sleep problems as our findings indicate. There are implications that poorer socio-economic status increases the risk for psychological stress based on findings related to previous infectious disease outbreaks, as well as that a socio-economic status is a potential influencing factor in elevating COVID-19 risk among those with mental health issues ^[39]^. Higher mean age of morning-types might also influence the prevalence of mood problems during the pandemic as higher age has been reported to be associated with lower mood problems amid the pandemic in China ^[5]^. However, evening-types also showed poorer mental health, and worse well-being, quality of life, and health than morning-types when age was controlled for. Yet, possibly due to this age difference, higher education and permanent job situation during the pandemic were more common among morning-types than among evening-types in our sample. Furthermore, evening-types had higher unemployment rate, suffered more financially due to pandemic as well as had been more often and longer in confinement than other circadian types. Unfortunately, we did not have information on the socio-economic or mental health status of the participants before the pandemic. However, our results show that definite evening-types had more sleep and mental health problems than other circadian types regardless of whether they had suffered financially or been in confinement during the pandemic, although these factors had a moderating role by increasing the risk for sleep and mental health problems among circadian types, and especially among definite evening-types. Besides additional potential contributing mechanisms, such as genetic ^[40]^, on higher risk for sleep and mental health problems among evening-types, it is likely that evening-types have been more susceptible to sleep problems during the pandemic due to their higher baseline occurrence of sleep problems as compared to other circadian types. Higher risk for sleep and mental health issues among evening-types as compared to morning-types have also been reported previously in large population-based adult samples ^[19–21, 41]^.

Interestingly, although evening-types showed the highest prevalence of impaired sleep amid the pandemic, compared to other circadian types these sleep problems decreased more for a subgroup of evening-types. This subgroup consisted of definite evening-types with the highest level of sleep problems before the pandemic. Increased flexibility of sleep–wake schedules might play a role in the positive effects on sleep during the pandemic especially among evening-types who have been able to adjust their sleep–wake schedule and sleep duration to better match their innate circadian rhythms. Similarly, previous studies have reported an increase in sleep duration amid the pandemic along with a delay in sleep–wake schedules especially on working days ^[9, 10]^. Notably, we showed that while evening-types had shorter nightly sleep duration before the pandemic than other circadian types, this difference disappeared amid the pandemic and the 24-hour sleep duration was significantly longer among evening-types versus morning-types. Thus daily naps were more common among evening-types than before. Our results showed that even though sleep–wake timing was delayed among all circadian types, this was most pronounced among evening-types and especially on working days, indicating adjustment of sleep–wake schedule towards preferred timing. The majority of definite evening-types with information on midpoint of sleep shifted their midpoint of sleep to a later-time, especially on working days, and more than every other evening-type showed also a delay in their midpoint of sleep on free days. A shift towards earlier-timed sleep–wake behavior during the pandemic was more common among definite morning-types than among other circadian types and especially on free days, although many of the definite morning-types showed a delay in sleep–wake rhythms. Our results thus indicate, that definite evening-types have been able to adjust their sleep–wake behavior more according to their circadian type during the pandemic especially on working days, while for some definite morning-types this has been more apparent regarding free days. In line with our findings, a recent study on 375 Australian athletes showed that evening-types reported more delay in their midpoint of sleep both on working days and on free days, longer sleep latency, as well as increased daytime sleepiness than other circadian types ^[26]^. However, no differences between the mental health score or stress were found in that study between the circadian types, which might relate to the smaller sample size and the target group consisting of young healthy athletes ^[26]^.

Sleep–wake behavior is affected by physical and environmental conditions, such as changes in time allocation and exposure to different environmental and social clues. Whether long-term effects on sleep–wake behavior due to the COVID-19 pandemic will be evident in coming years remains to be seen. Finding measures that could alleviate the increased health risks that have based on our findings accumulated especially to evening-types during the pandemic is crucial for preventing development of chronic health issues. One solution could be allowing more flexibility to societal schedules to better accommodate the different physiological needs regarding sleep–wake rhythms of different circadian types. If working remotely becomes a more common practice also after the pandemic as compared to the time before the pandemic, this can have beneficial effects especially for the well-being and work efficiency of evening-types, allowing them to adjust their sleep–wake behavior more according to their circadian type. Benefits of work flexibility should be examined more from the view-point of individual circadian type after the pandemic in order to tease apart the negative effects that a stressful and restricted time, such as the COVID-19 pandemic, has had on sleep and mental health.

The greatest strength of our study was the large multinational sample collected with a harmonized questionnaire making our study unique in assessment of sleep before and amid the pandemic globally. The major limitations to our study include collecting data cross-sectionally rather than longitudinally. There is thus a possibility for recall bias when reporting behavior before the pandemic. It could also be argued that administering the 19-item version of the Morningness/Eveningness questionnaire ^[42]^ rather than using a single item to assess circadian type would have been a more precise method of assessment. Nonetheless, a single morningness/eveningness item is commonly used in epidemiological research targeting large samples and has also been shown to be a fairly unchangeable character during adulthood ^[16]^, and not affected by age in adult population as much as behavioral midpoint of sleep on free days ^[28]^. Self-estimated circadian type also correlates better with the biological polygenetic score for morningness/eveningness in the general Finnish adult population as compared to habitual midpoint of sleep on free days ^[28]^. Further, consistent with the circadian typology ^[21, 43]^, sleep–wake behavior was progressively later-timed in our study and the average age was younger for those with circadian type more towards eveningness and vice versa for those with circadian type more towards morningness. In line with population-based adult samples from Finland, eveningness was more common in this multinational sample among women than among men ^[21]^, lending support to the generalizability of our findings.

## Conclusions

Overall, evening-types often struggle with sleep as their circadian type does not align with more morning-oriented societal schedules. Greater sleep/work schedule flexibility, e.g., due to work at home, as seen during the COVID-19 pandemic, may therefore allow better adjustment of sleep–wake behavior according to innate circadian type and increase sleep duration especially among evening-types. These benefits of the pandemic-related extension on sleep duration and on adjustment of sleep–wake behavior were, however, paralleled and over-weighted by an increased prevalence of sleep problems (e.g. insomnia symptoms, nightmares, and daytime sleepiness) and signs of lower mental health well-being, which was especially pronounced among definite evening-types. We found that confinement and having suffered financially due to the pandemic partly moderated these associations, however, financial suffering or confinement among evening-types did not completely explain the higher levels of problems compared to other circadian types. Other driving mechanisms contributing to the elevated sleep and mental health problems among evening-types may be innate, such as genetic ^[40]^ or related to disruption of circadian rhythms. It is also possible that evening-types have been more susceptible to sleep and mental health issues during the pandemic due to their higher occurrence of sleep problems already before the pandemic as compared to other circadian types. The findings presented here raise the concern of accumulation of a multitude of health problems in evening-types especially during the pandemic that might predispose to severe chronic conditions over time.

## Supplementary Material

zsab216_suppl_Supplementary_Figure_S1

zsab216_suppl_Supplementary_Materials

## Data Availability

The data will be shared on reasonable request to the corresponding author.
