# Supplementary figures and images for: Evening-types show highest increase of sleep and mental health problems during the COVID-19 pandemic—multinational study on 19 267 adults
Source: Sleep. 2021 Aug 25;45(2):zsab216. doi: 10.1093/sleep/zsab216 (PMC8499764; doi:10.1093/sleep/zsab216)

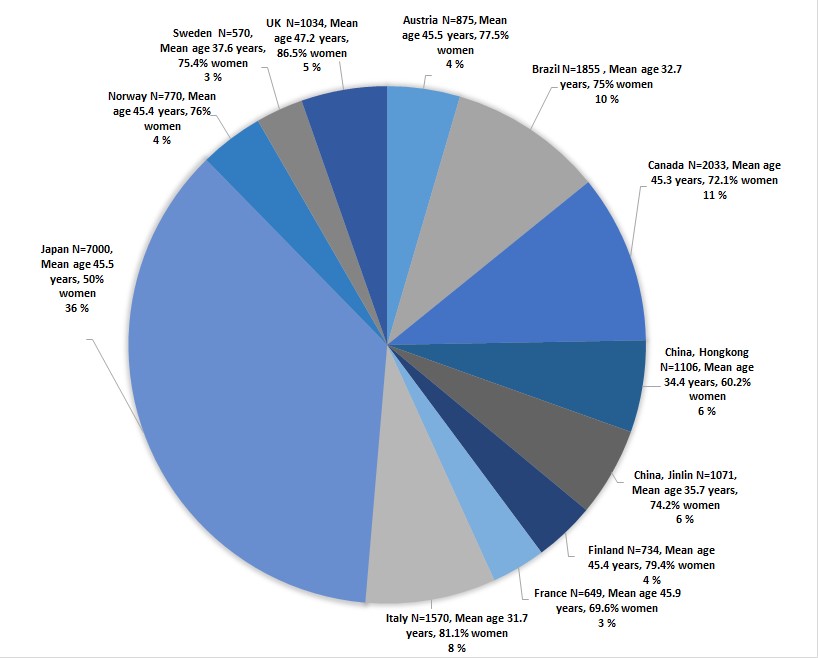

Supplement: zsab216_suppl_Supplementary_Figure_S1 [file zsab216_suppl_supplementary_figure_s1.jpeg]
