# Supplementary material for: Evening-types show highest increase of sleep and mental health problems during the COVID-19 pandemic—multinational study on 19 267 adults
Source: Sleep. 2021 Aug 25;45(2):zsab216. doi: 10.1093/sleep/zsab216 (PMC8499764; doi:10.1093/sleep/zsab216)
Supplement: zsab216_suppl_Supplementary_Materials [file zsab216_suppl_supplementary_materials.docx]

**Evening-types show highest increase of sleep and mental health problems during the COVID-19 pandemic – Multinational study on 19,267 adults**

Ilona Merikanto^1^, Laura Kortesoja^2^, Christian Benedict^3^, Frances Chung^4^, Jonathan Cedernaes^5^, Colin A. Espie^6^, Charles M. Morin^7^, Yves Dauvilliers^8^, Markku Partinen^9^, Luigi De Gennaro^10^, Yun Kwok Wing^11^, Ngan Yin Chan^12^, Yuichi Inoue^13^, Kentaro Matsui^14^, Brigitte Holzinger^15^, Giuseppe Plazzi^16^, Sérgio Arthuro Mota-Rolim ^17^, Damien Leger ^18^, Thomas Penzel^19^, Bjørn Bjorvatn^20^

^1^ SleepWell Research Program Unit, Faculty of Medicine, University of Helsinki, Helsinki, Finland; Department of Public Health Solutions, Finnish Institute for Health and Welfare, Helsinki, Finland; Orton Orthopaedics Hospital, Helsinki, Finland

^2^ Centre for Educational Assessment, University of Helsinki, 00014 Helsinki, Finland

^3^ Department of Neuroscience, Sleep Science (BMC), Uppsala University, Uppsala, Sweden

^4^ Department of Anesthesia and Pain Medicine, Toronto Western Hospital, University Health Network, University of Toronto, Toronto, Ontario, Canada; Institute of Medical Science, Temerty Faculty of Medicine, University of Toronto, Ontario, Canada

^5^ Department of Medical Sciences, Uppsala University, Uppsala, Sweden; Department of Medicine, Division of Endocrinology, Metabolism, and Molecular Medicine, Northwestern University, Chicago, IL, USA

^6^ Sleep and Circadian Neuroscience Institute, Nuffield Department of Clinical Neurosciences, University of Oxford, Oxford, UK

^7^ École de Psychologie, Centre d’étude des troubles du sommeil, Centre de recherche CERVO/Brain Research Center, Université Laval, Québec, Canada

^8^ Sleep-Wake Disorders Center, Department of Neurology, Gui-de-Chauliac Hospital, Institute for Neurosciences of Montpellier INM, INSERM, University of Montpellier, France

^9^ Helsinki Sleep Clinic, Vitalmed Research Center, and Department of Neurosciences, Clinicum, University of Helsinki, Helsinki, Finland

^10^ Department of Psychology, Sapienza University of Rome, Rome, Italy, and IRCCS Fondazione Santa Lucia, Rome, Italy

^11^ Departments of Psychiatry, Faculty of Medicine, The Chinese University of Hong Kong, Shatin, Hong Kong SAR, China

^12^ Li Chiu Kong Family Sleep Assessment Unit, Departments of Psychiatry, Faculty of Medicine, The Chinese University of Hong Kong, Shatin, Hong Kong SAR, China

^13^ Department of Somnology, Tokyo Medical University, Tokyo, Japan

^14^ Department of Laboratory Medicine, National Center Hospital, National Center of Neurology and Psychiatry, Tokyo, Japan

^15^ Institute for Dream and Consciousness Research; Medical University of Vienna, Austria

^16^ IRCCS – Institute of the Neurological Sciences of Bologna, Bologna, Italy; Department of Biomedical, Metabolic and Neural Sciences, University of Modena and Reggio Emilia, Modena, Italy

^17^ Brain Institute, Physiology and Behaviour Department, and Onofre Lopes University Hospital - Federal University of Rio Grande do Norte, Natal, Brazil

^18^ Hopital Hotel-Dieu de Paris, Sleep and Vigilance Center; Universite de Paris, VIFASOM (EA 7331 Vigilance Fatigue Sommeil et Santé Publique)

^19^ Sleep Medicine Center, Charite Universitätsmedizin Berlin, Berlin, Germany

^20^ Department of Global Public Health and Primary Care, University of Bergen, and Norwegian Competence Center for Sleep Disorders, Haukeland University Hospital, Bergen, Norway

*Correspondence concerning this article should be addressed to Ilona Merikanto, Research Programs Unit, Sleep and stress in health and in transition from acute to chronic diseases, Faculty of Medicine, PL21, 00014 University of Helsinki, Finland. E-mail: ilona.merikanto@helsinki.fi

**Supplementary Materials**

Supplementary Table 1. Sleep behavior and problems amid the pandemic by circadian type. Definite morning-types are defined as reference in the regression models. Model 1 is adjusted for age, sex, and duration of confinement during the pandemic and Model 2 for age, sex and socio-economic status during the pandemic. Regression estimate (B) and confidence intervals (95% CI) given in the Table. ***p<0.001, **p<0.01,*p<0.05.

|  | | **Definitive evening-types** | **Moderate evening-types** | **Intermediate-types** | **Moderate morning-types** |
| --- | --- | --- | --- | --- | --- |
|  | | B (95% CI) | B (95% CI) | B (95% CI) | B (95% CI) |
| **Sleep per night in hours** | |  |  |  |  |
|  | Model 1 | -0.08 (-0.2 ± 0.09) | 0.02 (-0.09 ± 01) | 0.06 (-0.2 ± 0.05) | 0.2 (0.05 ± 0.3)** |
|  | Model 2 | -0.08 (-0.2 ± 0.08) | 0.006 (-0.1 ± 0.1) | -0.1 (-0.2 ± 0.0001) | 0.1 (0.03 ± 0.2)* |
| **Sleep per 24h in hours** | |  |  |  |  |
|  | Model 1 | 0.4 (0.2 ± 0.6)*** | 0.1 (0.007 ± 0.3)* | 0.009 (-0.1 ± 0.1) | 0.1 (-0.02 ± 0.2) |
|  | Model 2 | 0.4 (0.2 ± 0.6)*** | 0.1 (-0.04 ± 0.2) | -0.09 (-0.2 ± 0.03) | 0.08 (-0.04 ± 0.2) |
| **Bedtime on working days** | | |  |  |  |
|  | Model 1 | 4843.5 (3927.5 ± 5759.5)*** | 1670.2 (807.8 ± 2532.6)*** | 633.1 (-298.9 ± 1565.0) | -312.3 (-1218.5 ± 593.9) |
|  | Model 2 | 4617.2 (3713.0 ± 5521.4)*** | 1329.7 (478.0 ± 2184.5)** | 303.8 (-594.2 ± 1201.7) | -560.2 (-1459.3 ± 338.9) |
| **Wake-up time at working days** | | |  |  |  |
|  | Model 1 | 7515.5 (6792.5 ± 8238.4)*** | 4312.3 (3832.9 ± 4791.7)*** | 2331.9 (1856.4 ± 2807.3)*** | 1222.1 (763.6 ± 1680.6)*** |
|  | Model 2 | 7461.1 (6758.9 ± 8163.4)*** | 4130.1 (3662.0 ± 4598.2)*** | 2136.2 (1675.7 ± 2596.7)*** | 1217.0 (775.6 ± 1658.5)*** |
| **Bedtime on free days** | |  |  |  |  |
|  | Model 1 | 4629.7 (3799.3 ± 5460.1)*** | 1721.1 (975.3 ± 2466.9)*** | 173.0 (-582.6 ± 928.6) | -733.2 (-1534.6 ± 68.2) |
|  | Model 2 | 4565.4 (3745.1 ± 5385.6)*** | 1469.4 (732.0 ± 2206.8)*** | -212.0 (-961.4 ± 537.4) | -850.0 (-1649.7 ± -50.2)* |
| **Wake-up time on free days** | | |  |  |  |
|  | Model 1 | 8794.1 (8060.9 ± 9527.3)*** | 5521.0 (5004.9 ± 6037.1)*** | 2719.4 (2215.0 ± 3223.9)*** | 1689.7 (1162.7 ± 2216.7)*** |
|  | Model 2 | 8993.7 (8268.4 ± 9719.0)*** | 5470.5 (4952.9 ± 5988.0)*** | 2435.7 (1929.2 ± 2942.2)*** | 1657.6 (1136.1 ± 2179.2)*** |
| **Midpoint of sleep at working days** | |  |  |  |  |
|  | Model 1 | 5712.9 (4794.5 ± 6631.4)*** | 2463.7 (1612.1 ± 3315.3)*** | 1142.8 (224.2 ± 2061.5)* | 311.3 (-578.1 ± 1200.7) |
|  | Model 2 | 5471.0 (4571.3 ± 6370.7)*** | 2006.3 (1165.4 ± 2847.1)*** | 670.9 (-211.9 ± 1553.7) | -32.6 (-912.1 ± 846.8) |
| **Midpoint of sleep at free days** | |  |  |  |  |
|  | Model 1 | 5977.1 (5240.8 ± 6713.5)*** | 2844.2 (2209.9 ± 3478.5)*** | 847.5 (187.7 ± 1507.2)* | -28.4 (-717.5 ± 660.7) |
|  | Model 2 | 6032.8 (5307.3 ± 6758.4)*** | 2628.7 (2002.5 ± 3254.9)*** | 403.4 (-246.4 ± 1053.1) | -116.7 (-801.6 ± 568.1) |
| **Poor sleep quality** | |  |  |  |  |
|  | Model 1 | 1.3 (1.2 ± 1.4)*** | 0.8 (0.7 ± 0.9)*** | 0.4 (0.3 ± 0.5)*** | 0.2 (0.1 ± 0.3)** |
|  | Model 2 | 0.8 (0.7 ± 0.9)*** | 0.4 (0.3 ± 0.5)*** | 0.2 (0.1 ± 0.4)*** | 0.1 (0.2 ± 0.3)* |
| **Sleep onset problems** | |  |  |  |  |
|  | Model 1 | 1.4 (1.2 ± 1.5)*** | 0.8 (0.7 ± 0.9)*** | 0.3 (0.1 ± 0.4)*** | 0.2 (0.06 ± 0.3)** |
|  | Model 2 | 1.4 (1.2 ± 1.5)*** | 0.8 (0.6 ± 0.9)*** | 0.3 (0.1 ± 0.4)*** | 0.2 (0.07 ± 0.3)** |
| **Sleep maintenance problems** | |  |  |  |  |
|  | Model 1 | 0.2 (0.02 ± 0.4)* | 0.07 (-0.06 ± 0.2) | -0.06 (-0.2 ± -0.06) | 0.00005 (-0.1 ± 0.1) |
|  | Model 2 | 0.3 (0.09 ± 0.4)** | 0.06 (-0.06 ± 0.2) | -0.1 (-0.2 ± -0.007) | 0.01 (-0.1 ± 0.1) |
| **Early morning awakening** | |  |  |  |  |
|  | Model 1 | -0.3 (-0.5 ± -0.2)*** | -0.3 (-0.4 ± -0.1)*** | -0.4 (-0.5 ± -0.2)*** | -0.1 (-0.2 ± 0.02) |
|  | Model 2 | -0.3 (-0.4 ± -0.09)** | -0.2 (-0.4 ± -0.1)*** | -0.4 (-0.5 ± -0.3)*** | -0.09 (-0.2 ± 0.04) |
| **Hypnotics use** | |  |  |  |  |
|  | Model 1 | 0.5 (0.2 ± 0.7)*** | 0.08 (-0.1 ± 0.3) | 0.01 (-0.2 ± 0.2) | -0.1 (-0.4 ± 0.07) |
|  | Model 2 | 0.5 (0.3 ± 0.7)*** | 0.6 (-0.2 ± 0.3) | -0.03 (-0.2 ± 0.2) | -0.1 (-0.4 ± 0.07) |
| **Excessive sleepiness** | |  |  |  |  |
|  | Model 1 | 1.0 (0.8 ± 1.2)*** | 0.6 (0.5 ± 0.7)*** | 0.2 (0.05 ± 0.3)** | 0.1 (0.01 ± 0.3)* |
|  | Model 2 | 1.0 (0.9 ± 1.2)*** | 0.6 (0.4 ± 0.7)*** | 0.1 (-0.02 ± 0.2) | 0.2 (0.03 ± 0.3)* |
| **Fatigue** | |  |  |  |  |
|  | Model 1 | 0.8 (0.7 ± 1.0)*** | 0.4 (0.3 ± 0.6)*** | 0.03 (-0.09 ± 0.2) | 0.07 (-0.05 ± 0.2) |
|  | Model 2 | 0.9 (0.7 ± 1.0)*** | 0.4 (0.3 ± 0.6)*** | -0.02 (-0.1 ± 0.1) | 0.08 (-0.03 ± 0.2) |
| **Nightmares** | |  |  |  |  |
|  | Model 1 | 0.5 (0.4 ± 0.7)*** | 0.3 (0.2 ± 0.4)*** | 0.1 (-0.01 ± 0.2) | 0.1 (-0.04 ± 0.2) |
|  | Model 2 | 0.5 (0.4 ± 0.7)*** | 0.3 (0.2 ± 0.4)*** | 0.06 (-0.07 ± 0.2) | 0.1 (-0.02 ± 0.2) |
| **Insomnia severity index (ISI) amid pandemic** | |  |  |  |  |
|  | Model 1 | 0.8 (0.6 ± 1.0)*** | 0.3 (0.2 ± 0.4)*** | -0.06 (-0.2 ± 0.07) | -0.07 (-0.2 ± 0.07) |
|  | Model 2 | 0.9 (0.8 ± 1.1)*** | 0.3 (0.2 ± 0.4)*** | -0.2 (-0.3 ± -0.03)* | -0.05 (-0.2 ± 0.09) |
| **Anxiety past two weeks** | |  |  |  |  |
|  | Model 1 | 0.5 (0.3 ± 0.6)*** | 0.1 (-0.05 ± 0.3) | -0.2 (-0.3 ± -0.001)* | -0.2 (-0.4 ± -0.06)** |
|  | Model 2 | 0.6 (0.4 ± 0.8)*** | 0.1 (-0.03 ± 0.3) | -0.3 (-0.4 ± -0.1)* | -0.2 (-0.3 ± -0.01)* |
| **Depression past two weeks** | |  |  |  |  |
|  | Model 1 | 0.8 (0.6 ± 0.9)*** | 0.3 (0.2 ± 0.5)*** | 0.07 (-0.1 ± 0.2) | 0.04 (-0.1 ± 0.2) |
|  | Model 2 | 0.9 (0.7 ± 1.1)*** | 0.3 (0.1 ± 0.5)*** | -0.08 (-0.3 ± 0.1) | 0.05 (-0.1 ± 0.2) |
| **Well-being index past two weeks** | |  |  |  |  |
|  | Model 1 | -13.7 (-15.6 ± -11.9)*** | -8.9 (-10.4 ± -7.4)*** | -4.2 (-5.7 ± -2.7)*** | -3.4 (-4.9 ± -1.8)*** |
|  | Model 2 | -14.8 (-16.7 ± -13.0)*** | -8.8 (-10.4 ± -7.3)*** | -3.3 (-4.8 ± -1.8)*** | -3.6 (-5.2 ± -2.1)*** |
| **PTSD symptoms amid pandemic: Repeated disturbing thoughts and memories** | |  |  |  |  |
|  | Model 1 | 0.7 (0.6 ± 0.9)*** | 0.3 (0.2 ± 0.4)*** | 0.1 (-0.02 ± 0.2) | 0.03 (-0.1 ± 0.2) |
|  | Model 2 | 0.8 (0.6 ± 0.9)*** | 0.3 (0.2 ± 0.4)*** | 0.02 (-0.01 ± 0.2) | 0.03 (-0.1 ± 0.2) |
| **PTSD symptoms amid pandemic: Feeling very upset of past** | |  |  |  |  |
|  | Model 1 | 0.7 (0.5 ± 0.8)*** | 0.3 (0.1 ± 0.4)*** | 0.1 (-0.02 ± 0.2) | 0.02 (-0.1 ± 0.2) |
|  | Model 2 | 0.7 (0.6 ± 0.9)*** | 0.2 (0.1 ± 0.4)*** | 0.01 (-0.01 ± 0.1) | 0.02 (-0.1 ± 0.2) |
| **Stress at the time of response** | |  |  |  |  |
|  | Model 1 | 0.8 (0.6 ± 0.9)*** | 0.4 (0.2 ± 0.5)*** | 0.06 (-0.06 ± 0.2) | 0.08 (-0.04 ± 0.2) |
|  | Model 2 | 0.8 (0.7 ± 0.9)*** | 0.4 (0.2 ± 0.5)*** | -0.03 (-0.03 ± 0.2) | 0.09 (-0.03 ± 0.2) |
| **Quality of life at the time of response** | |  |  |  |  |
|  | Model 1 | -8.6 (-10.6 ± -6.6)*** | -3.0 (-4.5 ± -1.5)*** | -2.3 (-3.8 ± -0.9)** | 0.3 (-1.2 ± 1.8) |
|  | Model 2 | -8.0 (-10.0 ± -6.1)*** | -2.5 (-4.0 ± -1.0)*** | -2.3 (-3.8 ± -0.9)** | 0.2 (-1.3 ± 1.7) |
| **Quality of health at the time of response** | |  |  |  |  |
|  | Model 1 | -7.1 (-9.1 ± -5.1)*** | -3.6 (-5.1 ± -2.1)*** | -2.3 (-3.7 ± 0.8)** | -0.3 (-1.8 ± 1.2) |
|  | Model 2 | -7.2 (-9.1 ± -5.2)*** | -3.3 (-4.8 ± -1.8)*** | -2.1 (-3.6 ± 0.6)** | -0.3 (-1.8 ± 1.2) |

Supplementary Table 2. Moderation analyses with either sufferance to financial status or confinement as moderators between the association of circadian type and sleep problems amid the pandemic. Definite morning-types and no sufferance to financial status/not being in confinement are defined as reference (*REF*) in the regression models, adjusted for age and sex. ***p<0.001, **p<0.01,*p<0.05.

|  | | **Definitive evening-types** | **Moderate evening-types** | **Intermediate-types** | **Moderate morning-types** | **Definite morning-types** |
| --- | --- | --- | --- | --- | --- | --- |
|  | | B (95%CI) | B (95%CI) | B (95%CI) | B (95%CI) | B (95%CI) |
| **Poor sleep quality** | |  |  |  |  |  |
|  | Suffered financially: Yes | 1.0 (0.8 ± 1.2)*** | 0.5 (0.3 ± 0.6)*** | 0.4 (0.2 ± 0.6)*** | 0.2 (0.06 ± 0.4)** | 0.2 (-0.03 ± 0.4) |
|  | Suffered financially: No | 0.6 (0.4 ± 0.9)*** | 0.4 (0.2 ± 0.6)*** | 0.1 (-0.04 ± 0.3) | 0.2 (-0.04 ± 0.3) | *REF* |
|  | Confinement: Yes | 0.9 (0.7 ± 1.1)*** | 0.5 (0.3 ± 0.6)*** | 0.3 (0.1 ± 0.5)*** | 0.1 (-0.02 ± 0.3) | 0.1 (-0.06 ± 0.3) |
|  | Confinement: No | 0.5 (0.3 ± 0.8)*** | 0.4 (0.2 ± 0.5)*** | 0.2 (0.09 ± 0.4)*** | 0.2 (0.08 ± 0.4)** | *REF* |
| **Sleep onset problems** | |  |  |  |  |  |
|  | Suffered financially: Yes | 1.7 (1.5 ± 1.9)*** | 1.1 (0.9 ±1.2)*** | 0.4 (0.2 ± 0.6)*** | 0.4 (0.2 ± 0.5)*** | 0.1 (-0.06 ± 0.3) |
|  | Suffered financially: No | 1.3 (1.1 ± 1.5)*** | 0.8 (0.6 ± 1.0)*** | 0.2 (-0.006 ± 0.4) | 0.3 (0.1 ± 0.5)** | *REF* |
|  | Confinement: Yes | 2.1 (1.9 ± 2.3)*** | 1.6 (1.5 ± 1.8)*** | 1.1 (0.9 ± 1.3)*** | 0.9 (0.7 ± 1.0)*** | 0.7 (0.6 ± 0.9)*** |
|  | Confinement: No | 1.3 (1.1 ± 1.6)*** | 0.8 (0.6 ± 0.9)*** | 0.3 (0.2 ± 0.4)*** | 0.4 (0.2 ± 0.5)*** | *REF* |
| **Sleep maintenance problems** | | |  |  |  |  |
|  | Suffered financially: Yes | 0.2 (-0.005 ± 0.4) | 0.04 (-0.1 ± 0.2) | -0.2 (-0.3 ± 0.005) | -0.04 (-0.2 ± 0.1) | 0.004 (-0.02 ± 0.2) |
|  | Suffered financially: No | -0.002 (-0.2 ± 0.2) | -0.2 (-0.4 ± -0.006)* | -0.2 (-0.4 ± -0.02)* | -0.09 ( -0.3 ± 0.09) | *REF* |
|  | Confinement: Yes | 0.4 (0.3 ± 0.6)*** | 0.3 (0.1 ± 0.4)*** | 0.3 (0.07 ± 0.4)** | 0.2 (0.04 ± 0.4)* | 0.4 (0.2 ± 0.5)*** |
|  | Confinement: No | -0.005 (-0.2 ± 0.2) | -0.03 (-0.2 ± 0.1) | -0.1 (-0.3 ± 0.002) | 0.08 (-0.07 ± 0.2) | *REF* |
| **Early morning awakening** | | |  |  |  |  |
|  | Suffered financially: Yes | -0.2 (-0.5 ± -0.02)* | -0.2 (-0.4 ± -0.07)** | -0.4 (-0.6 ± -0.2)*** | -0.1 (-0.3 ± 0.08) | 0.008 (-0.2 ± 0.2) |
|  | Suffered financially: No | -0.5 (-0.8 ± -0.3)*** | -0.5 (-0.7 ± -0.3)*** | -0.5 (-0.7 ± -0.3)*** | -0.2 (-0.4 ± -0.03)* | *REF* |
|  | Confinement: Yes | 0.01 (-0.2 ± 0.2) | 0.09 (-0.07 ± 0.3) | 0.1 (-0.03 ± 0.3) | 0.3 (0.09 ± 0.4)** | 0.6 (0.4 ± 0.7)*** |
|  | Confinement: No | -0.2 (-0.4 ± 0.04) | -0.2 (-0.4 ± -0.07)** | -0.4 (-0.5 ± -0.2)*** | 0.03 (-0.1 ± 0.2) | *REF* |
| **Hypnotics use** | |  |  |  |  |  |
|  | Suffered financially: Yes | 0.7 (0.4 ± 1.0)*** | 0.3 (0.04 ± 0.6)* | 0.1 (-0.2 ± 0.4) | -0.05 (-0.4 ± 0.3) | 0.1 (-0.2 ± 0.4) |
|  | Suffered financially: No | 0.4 (0.02 ± 0.7)* | -0.3 (-0.6 ± 0.05) | -0.09 (-0.4 ± 0.2) | -0.1 (-0.5 ± 0.2) | *REF* |
|  | Confinement: Yes | 0.8 (0.5 ± 1.1)*** | 0.5 (0.2 ± 0.7)*** | 0.4 (0.1 ± 0.7)** | 0.1 (-0.2 ± 0.4) | 0.5 (0.2 ± 0.8)*** |
|  | Confinement: No | 0.7 (0.4 ± 1.1)*** | 0.2 (-0.1 ± 0.4) | 0.1 (-0.1 ± 0.3) | 0.2 (-0.04 ± 0.5) | *REF* |
| **Excessive sleepiness** | | |  |  |  |  |
|  | Suffered financially: Yes | 1.5 (1.3 ± 1.7)*** | 0.8 (0.7 ± 1.0)*** | 0.3 (0.1 ± 0.5)*** | 0.4 (0.2 ± 0.5)*** | 0.2 (-0.03 ± 0.4) |
|  | Suffered financially: No | 0.9 (0.7 ±1.2)*** | 0.6 (0.4 ± 0.8)*** | 0.01 (-0.2 ± 0.2) | 0.2 (0.003 ± 0.4)* | *REF* |
|  | Confinement: Yes | 1.7 (1.5 ± 1.8)*** | 1.2 (1.0 ± 1.3)*** | 0.7 (0.6 ± 0.9)*** | 0.6 (0.5 ± 0.8)*** | 0.5 (0.4 ± 0.7)*** |
|  | Confinement: No | 0.9 (0.7 ± 1.2)*** | 0.6 (0.5 ± 0.8)*** | 0.1 (0.009 ± 0.3)* | 0.4 (0.2 ± 0.5)*** | *REF* |
| **Fatigue** | |  |  |  |  |  |
|  | Suffered financially: Yes | 1.3 (1.0 ± 1.5)*** | 0.7 (0.5 ± 0.8)*** | 0.1 (-0.04 ± 0.3) | 0.2 (0.05 ± 0.4)* | 0.2 (-0.03 ± 0.3) |
|  | Suffered financially: No | 0.7 (0.5 ± 0.9)*** | 0.5 (0.3 ± 0.6)*** | -0.1 (-0.3 ±0.07) | 0.2 ( 0.01 ± 0.4)* | *REF* |
|  | Confinement: Yes | 1.5 (1.3 ± 1.7)*** | 1.1 (0.9 ± 1.2)*** | 0.6 (0.4 ± 0.8)*** | 0.6 (0.4 ± 0.7)*** | 0.6 (0.5 ±0.8)*** |
|  | Confinement: No | 0.9 (0.6 ±1.1)*** | 0.5 (0.4 ± 0.7)*** | 0.1 (-0.04 ± 0.2) | 0.4 (0.2 ± 0.5)*** | *REF* |
| **Nightmares** | |  |  |  |  |  |
|  | Suffered financially: Yes | 1.2 (0.9 ± 1.4)*** | 0.8 (0.6 ± 1.0)*** | 0.3 (0.1 ± 0.5)*** | 0.4 (0.2 ± 0.6)*** | 0.2 (0.04 ± 0.4)* |
|  | Suffered financially: No | 0.7 (0.4 ± 0.9)*** | 0.3 (0.2 ± 0.5)*** | 0.08 (-0.1 ± 0.3) | 0.3 (0.1 ± 0.5)** | *REF* |
|  | Confinement: Yes | 1.3 (1.1 ± 1.5)*** | 1.0 (0.9 ± 1.2)*** | 0.7 (0.6 ± 0.9)*** | 0.7 (0.5 ± 0.9)*** | 0.6 (0.4 ± 0.8)*** |
|  | Confinement: No | 0.6 (0.4 ± 0.8)*** | 0.4 (0.3 ± 0.6)*** | 0.09 (-0.05 ± 0.2) | 0.3 (0.1 ± 0.4)*** | *REF* |
| **Insomnia severity index (ISI)** | |  |  |  |  |  |
|  | Suffered financially: Yes | 1.4 (1.2 ± 1.6)*** | 0.6 (0.4 ± 0.8)*** | 0.04 (-0.2 ± 0.2) | 0.2 (-0.05 ± 0.3) | 0.1 (-0.1 ± 0.3) |
|  | Suffered financially: No | 0.7 (0.4 ± 0.9)*** | 0.2 (0.02 ± 0.4)* | -0.4 (-0.6 ± .0.2)*** | -0.04 (-0.3 ± 0.2) | *REF* |
|  | Confinement: Yes | 1.8 (1.6 ± 2.0)*** | 1.3 (1.1 ± 1.5)*** | 0.9 (0.7 ± 1.1)*** | 0.8 (0.6 ± 1.0)*** | 0.9 (0.7 ±1.1)*** |
|  | Confinement: No | 0.8 (0.6 ± 1.1)*** | 0.3 (0.1 ± 0.5)*** | -0.1 (-0.3 ± 0.04) | 0.09 (-0.07 ± 0.3) | *REF* |

|  | | | **Confinement**  No / Yes % | | p-value | | **Financial status suffered**  No / A little or somewhat / Much or severely % | | p-value | | **Baseline sleep before the pandemic** Mean hh.mm /% sleep problems ≥3 days/nights per week | p-value | **Sleep during the pandemic**  Mean hh.mm /% sleep problems ≥3 days/nights per week | p-value |
| --- | --- | --- | --- | --- | --- | --- | --- | --- | --- | --- | --- | --- | --- | --- |
| **Sleep per night in hours** | | | |  | | |  | |  | |  |  |  |  |
|  | Decreased | | 19.0 / 81.0 | | <0.001 | | 36.2 / 46.2 / 17.6 | | <0.01 | | 7h 23min | <0.001 | 5h 9min | <0.001 |
|  | Unchanged | | 39.2 / 60.8 | |  | | 44.5 / 43.6 /11.9 | |  | | 6h 53min |  | 6h 53min |  |
|  | Increased | | 15.3 / 84.7 | |  | | 36.6 / 52.8 / 10.7 | |  | | 6h 35min |  | 8h 47min |  |
| **Sleep maintenance problems** | | | |  | |  | |  | |  | |  |  |  |
|  | Decreased | | 16.9 / 83.1 | | <0.001 | | 39.8 / 49.5 / 10.7 | | 0.3 | | 38.1 | <0.001 | 8.6 | <0.001 |
|  | Unchanged | | 27.9 / 69.1 | |  | | 39.7 / 48.3 / 12.1 | |  | | 21.4 |  | 21.4 |  |
|  | Increased | | 13.9 / 86.1 | |  | | 35.6 /48.5 / 15.9 | |  | | 8.1 |  | 60.4 |  |
| **Early morning awakening** | | | |  | | |  | |  | |  |  |  |  |
|  | Decreased | | 14.7 / 85.3 | | <0.001 | | 40.5 / 48.2 / 11.3 | | 0.1 | | 25.6 | <0.001 | 1.2 | <0.001 |
|  | Unchanged | | 27.9 / 72.1 | |  | | 39.2 / 49.4 / 11.5 | |  | | 11.3 |  | 11.3 |  |
|  | Increased | | 14.6 / 85.4 | |  | | 36.7 / 45.9 / 17.4 | |  | | 6.5 |  | 44.6 |  |
| **Hypnotics use** | |  | |  | |  | |  | |  | |  |  |  |
|  | Decreased | | 12.4 / 87.6 | | <0.05 | | 52.8 / 36.5 / 10.7 | | 0.3 | | 39.2 | <0.001 | 0 | <0.001 |
|  | Unchanged | | 23.0 / 77.0 | |  | | 38.6 / 48.4 / 13.0 | |  | | 6.1 |  | 6.1 |  |
|  | Increased | | 15.2 / 84.8 | |  | | 33.0 / 50.3 / 16.7 | |  | | 5.0 |  | 49.9 |  |
| **Excessive sleepiness** | | | |  | |  | |  | |  | |  |  |  |
|  | Decreased | | 17.1 / 82.9 | | <0.001 | | 36.1 / 50.2 / 13.7 | | 0.7 | | 52.4 | <0.001 | 8.3 | <0.001 |
|  | Unchanged | | 31.1 / 68.9 | |  | | 40.7 / 47.0 / 12.3 | |  | | 35.9 |  | 35.9 |  |
|  | Increased | | 13.4 / 86.6 | |  | | 37.3 / 48.5 / 14.2 | |  | | 12.4 |  | 75.5 |  |
| **Fatigue** | |  | |  | |  | |  | |  | |  |  |  |
|  | Decreased | | 16.7 / 83.3 | | <0.001 | | 40.0 / 48.8 / 11.2 | | 0.5 | | 56.0 | <0.001 | 5.2 | <0.001 |
|  | Unchanged | | 32.8 / 67.2 | |  | | 40.7 / 46.1 / 13.2 | |  | | 40.0 |  | 40.0 |  |
|  | Increased | | 13.7 / 86.3 | |  | | 35.7 / 49.9 / 14.5 | |  | | 12.3 |  | 77.5 |  |
| **Nightmares** | |  | |  | |  | |  | |  | |  |  |  |
|  | Decreased | | 15.9 / 84.1 | | <0.001 | | 36.6 / 46.2 / 17.3 | | <0.01 | | 35.3 | <0.001 | 3.6 | <0.001 |
|  | Unchanged | | 29.5 / 70.5 | |  | | 43.4 / 43.6 / 13.0 | |  | | 5.7 |  | 5.7 |  |
|  | Increased | | 12.4 / 87.6 | |  | | 32.1 / 54.9 /12.9 | |  | | 2.7 |  | 39.7 |  |

Supplementary Table 3. Descriptive information on pandemic effects, baseline sleep before the pandemic and sleep amid the pandemic among definite evening-types by change in sleep problems. Differences between circadian types were analyzed with chi-square tests within SPSS Complex Samples Crosstabs and continuous measurements with t-test within SPSS Complex Samples General Linear Model (CSGLM).

Supplementary Table 4. Moderation analyses with either sufferance to financial status or confinement as moderators between the association of circadian type and mental health amid the pandemic. Definite morning-types and no sufferance to financial status/not being in confinement are defined as reference in the regression models, adjusted for age and sex. ***p<0.001, **p<0.01,*p<0.05.

|  | | **Definitive evening-types** | **Moderate evening-types** | **Intermediate-types** | **Moderate morning-types** | **Definite morning-types** |
| --- | --- | --- | --- | --- | --- | --- |
|  | | B (95%CI) | B (95%CI) | B (95%CI) | B (95%CI) | B (95%CI) |
| **Anxiety past two weeks** | |  |  |  |  |  |
|  | Suffered financially: Yes | 1.3 (1.1 ± 1.6)*** | 0.7 (0.5 ± 0.9)*** | 0.001 (-0.2 ± 0.2) | 0.2 (-0.07 ± 0.4) | 0.3 (0.02 ± 0.5)* |
|  | Suffered financially: No | 0.5 (0.2 ± 0.8)*** | 0.2 (-0.02 ± 0.5) | -0.3 (-0.5 ± -0.07)* | 0.04 (-0.2 ± 0.3) | *REF* |
|  | Confinement: Yes | 2.1 (1.9 ± 2.3)*** | 1.8 (1.6 ± 2.0)*** | 1.4 (1.1 ± 1.6)*** | 1.3 (1.1 ± 1.5)*** | 1.5 (1.3 ± 1.8)*** |
|  | Confinement: No | 0.9 (0.6 ± 1.2)*** | 0.4 (0.1 ±0.6)** | -0.05 (-0.3 ± 0.2) | 0.2 (-0.06 ± 0.4) | *REF* |
| **Depression past two weeks** | |  |  |  |  |  |
|  | Suffered financially: Yes | 1.7 (1.4 ± 1.9)*** | 1.0 (0.7 ± 1.2)*** | 0.2 (-0.005 ± 0.5) | 0.5 (0.2 ± 0.7)*** | 0.3 (0.04 ± 0.6)* |
|  | Suffered financially: No | 0.8 (0.5 ± 1.1)*** | 0.3 (0.08 ± 0.6)* | -0.01 (-0.3 ± 0.3) | 0.2 (-0.05 ± 0.5) | *REF* |
|  | Confinement: Yes | 2.3 (2.0 ± 2.5)*** | 1.8 (1.6 ± 2.1)*** | 1.5 (1.2 ± 1.7)*** | 1.4 (1.2 ± 1.7)*** | 1.3 (1.1 ± 1.6)*** |
|  | Confinement: No | 1.2 (0.9 ± 1.5)*** | 0.6 (0.3 ± 0.8)*** | 0.09 (-0.2 ± 0.3) | 0.4 (0.1 ± 0.6)** | *REF* |
| **Well-being index past two weeks** | | |  |  |  |  |
|  | Suffered financially: Yes | -22.7 (-25.2 ± -20.2)*** | -15.1 (-17.3 ± -13.0)*** | -7.9 (-10.1 ± -5.8)*** | -7.7 (-9.9 ± -5.5)*** | -4.7 (-7.2 ± -2.3)*** |
|  | Suffered financially: No | -15.8 (-18.7 ± -12.9)*** | -10.9 (-13.2 ± -8.6)*** | -2.7 (-5.0 ± -0.4)* | -6.6 (-9.0 ± -4.3)*** | *REF* |
|  | Confinement: Yes | -25.4 (-27.5 ± -23.2)*** | -20.1 (-21.9 ± -18.2)*** | -14.5 (-16.7 ± -12.4)*** | -12.8 (-14.8 ± -10.8)*** | -10.5 (-12.8 ± -8.2)*** |
|  | Confinement: No | -17.5 (-20.2 ± -14.8)*** | -11.4 (-13.2 ± -9.6)*** | -4.7 (-6.4 ± -3.1)*** | -7.2 (-9.0 ± -5.5)*** | *REF* |
| **Repeated disturbing thoughts and memories** | | |  |  |  |  |
|  | Suffered financially: Yes | 1.5 (1.3 ± 1.7)*** | 0.9 (0.8 ± 1.1)*** | 0.5 (0.3 ± 0.7)*** | 0.5 (0.4 ± 0.7)*** | 0.4 (0.2 ± 0.6)*** |
|  | Suffered financially: No | 0.9 (0.7 ± 1.1)*** | 0.4 (0.2 ± 0.6)*** | 0.01 (-0.2 ± 0.2) | 0.2 (0.01 ± 0.4)* | *REF* |
|  | Confinement: Yes | 1.7 (1.5 ± 1.9)*** | 1.3 (1.1 ± 1.4)*** | 1.0 (0.8 ±1.2)*** | 0.8 (0.7 ±1.0)*** | 0.8 (0.6 ± 1.0)*** |
|  | Confinement: No | 1.0 (0.7 ± 1.2)*** | 0.5 (0.4 ±0.7)*** | 0.2 (0.01 ± 0.3)* | 0.3 (0.2 ± 0.5)*** | *REF* |
| **Feeling very upset of past** | |  |  |  |  |  |
|  | Suffered financially: Yes | 1.4 (1.2 ± 1.6)*** | 0.8 (0.7 ± 1.0)*** | 0.4 (0.3 ± 0.6)*** | 0.5 (0.3 ± 0.7)*** | 0.4 (0.2 ± 0.6)*** |
|  | Suffered financially: No | 0.8 (0.6 ± 1.1)*** | 0.3 (0.1 ± 0.5)** | -0.04 (-0.2 ± 0.2) | 0.2 (-0.05 ± 0.3) | *REF* |
|  | Confinement: Yes | 1.6 (1.5 ± 1.8)*** | 1.2 (1.1 ± 1.4)*** | 1.0 (0.9 ± 1.2)*** | 0.8 (0.7 ± 1.0)*** | 0.8 (0.6 ± 1.0)*** |
|  | Confinement: No | 0.9 (0.7 ± 1.1)*** | 0.4 (0.3 ± 0.6)*** | 0.05 (-0.09 ± 0.2) | 0.3 (0.1 ± 0.4)** | *REF* |
| **Stress** | | |  |  |  |  |
|  | Suffered financially: Yes | 1.4 (1.2 ± 1.6)*** | 0.8 (0.6 ± 1.0)*** | 0.2 (0.06 ± 0.4)** | 0.4 (0.2 ± 0.6)*** | 0.3 (0.07 ± 0.5)** |
|  | Suffered financially: No | 0.8 (0.6 ± 1.0)*** | 0.4 (0.2 ± 0.6)*** | -0.1 (-0.3 ± 0.07) | 0.2 (0.06 ± 0.4)* | *REF* |
|  | Confinement: Yes | 1.8 (1.6 ± 2.0)*** | 1.4 (1.2 ± 1.5)*** | 1.1 (0.9 ± 1.2)*** | 1.0 (0.8 ± 1.1)*** | 1.0 (0.8 ± 1.2)*** |
|  | Confinement: No | 1.0 (0.7 ± 1.2)*** | 0.6 (0.4 ± 0.7)*** | 0.04 (-1.0 ± 0.2) | 0.4 (0.3 ± 0.5)*** | *REF* |
| **Quality of life** | |  |  |  |  |  |
|  | Suffered financially: Yes | -18.7 (-21.2 ± -16.2)*** | -11.7 (-13.6 ± -9.9)*** | -10.8 (-12.7 ± -8.9)*** | -7.7 (-9.6 ± -5.8)*** | -8.9 (-11.1 ± -6.7)*** |
|  | Suffered financially: No | -8.3 (-10.9 ± -5.7)*** | -4.3 (-6.3 ± -2.3)*** | -3.3 (-5.3 ± -1.4)*** | -2.2 (-4.2 ± -0.2)* | *REF* |
|  | Confinement: Yes | -9.9 (-12.2 ± -7.5)*** | -3.9 (-5.7 ± -2.0)*** | -2.6 (-4.6 ± -0.5)* | 0.8 (-1.1 ± 2.7) | -0.5 (-2.8 ± 1.7) |
|  | Confinement: No | -11.2 (-14.3 ± -8.1)*** | -5.3 (-7.1 ± -3.5)*** | -3.4 (-5.1 ± -1.8)*** | -2.9 (-4.7 ± -1.1)** | *REF* |
| **Quality of health** | |  |  |  |  |  |
|  | Suffered financially: Yes | -13.4 (-16.0 ± -10.8)*** | -8.4 (-10.3 ± -6.5)*** | -7.7 (-9.6 ± -5.8)*** | -4.7 (-6.6 ± -2.7)*** | -5.5 (-7.8 ± -3.3)*** |
|  | Suffered financially: No | -5.7 (-8.3 ± -3.0)*** | -3.9 (-5.9 ± -1.9)*** | -1.9 (-3.8 ± 0.03) | -1.9 (-3.8 ± 0.1) | *REF* |
|  | Confinement: Yes | -7.9 (-10.3 ± -5.5)*** | -4-0 (-5.9 ± -2.2)*** | -2.3 (-4.5 ± -0.2)* | 0.6 (-1.3 ± 2.5) | -0.1 (-2.4 ± 2.2) |
|  | Confinement: No | -6.3 (-8.8 ± -3.8)*** | -3.5 (-5.2 ± -1.7)*** | -2.3 (-3.9 ± -0.6)** | -2.2 (-4.0 ± -0.4)* | *REF* |

**Supplementary Figure legend**

Supplementary Figure 1. Information on the analytic sample by country.
